# Longitudinal Pilot Study of Progressive Urethral Sub-Obstruction in a Canine Model: Bladder Functional and Structural Changes and Exploratory Evaluation of Autologous Mesenchymal Stem Cells

**DOI:** 10.3390/vetsci13050460

**Published:** 2026-05-09

**Authors:** Mathilde Porato, Stéphanie Noël, Nadine Antoine, Géraldine Bolen, Joël Pincemail, Mutien-Marie Garigliany, Jean de Leval, Joëlle Piret, Frédéric Decortis, Annick Hamaide

**Affiliations:** 1 Department of Companion Animal Teaching and Clinical Practice, School of Veterinary Medicine, University of Liège, 4000 Liège, Belgium; 2 Department of Morphology and Pathology, School of Veterinary Medicine, University of Liège, 4000 Liège, Belgium; 3 Department of Cardiovascular Surgery, University Hospital, Boulevard de l’Hôpital, 4000 Liège, Belgium; 4 Department of Clinical Sciences, University Hospital, Boulevard de l’Hôpital, 4000 Liège, Belgium

**Keywords:** canine, urinary bladder, urethral obstruction, urodynamics, telemetry, stem cells, histology, RNA expression, immunostaining, oxidative stress

## Abstract

Bladder outlet obstruction (BOO) may lead to *detrusor* decompensation through progressive bladder remodeling. Most experimental studies rely on acutely induced BOO in rodents. Since progressive obstruction better reflects the condition, non-lethal models are needed to investigate chronic obstruction pathophysiology and evaluate regenerative therapies. This exploratory study aimed to evaluate (1) a progressive BOO model induced by an artificial urethral sphincter (AUS) in 2 dogs and (2) the systemic administration of autologous adipose-derived mesenchymal stem cells (ADMSCs) after obstruction release. Two intact male dogs underwent progressive BOO through gradual AUS inflation. Longitudinal assessment included telemetric urodynamic monitoring, urethral pressure profilometry, ultrasonography, post-void residual measurement, oxidative stress markers in serial blood samples and serial bladder biopsies for histology, transmission electron microscopy, immunohistochemistry, RT-qPCR and RNA sequencing (CCL2, CCR2, GFAP, VEGF, HGF). After AUS removal, one dog received three intravenous injections of 20 × 10^6^ PKH26-labelled autologous ADMSCs. BOO induced functional changes (increased *detrusor* pressure and urethral resistance, decreased urinary flow, prolonged voiding). No *detrusor* decompensation or fibrosis comparable to the human condition developed, encouraging refinement of this model. ADMSCs appeared to reach the bladder wall safely, but any influence on the glutathione redox system and CCL2 protein expression needs to be confirmed.

## Simple Summary

This study explored whether slowly narrowing the urinary outlet in two male dogs could reproduce bladder damage seen in men with long-standing prostate enlargement and whether the dogs’ own stem cells could aid recovery. When urine flow is blocked, the bladder must work harder, which can cause thickening, scarring and loss of function, reducing quality of life and increasing the risk of urinary infections and kidney problems. Most animal models use rodents and do not reflect the timing of these changes in humans. We gradually created a partial urethral blockage in two dogs using an adjustable device and monitored bladder function, structure and biological signals over time. Both dogs had temporary difficulty urinating and functional changes, but severe, irreversible damage did not occur. After removing the blockage, one dog received stem cells intravenously. Bladder function improved in both dogs. Stem cell treatment was safe to perform. Observed changes related to tissue repair and oxidative stress in the treated dog are preliminary and cannot be clearly attributed to the stem cells. While this model did not fully reproduce advanced bladder damage, the study suggests that stem cell therapy appears safe and encourages further investigation of regenerative treatments for chronic bladder dysfunction.

## 1. Introduction

Urinary bladder dysfunction due to urethral sub-obstruction is a common disorder in men. More specifically, bladder outlet obstruction corresponds to any disorder resulting in a reduction of urinary flow and an increase in *detrusor* pressure [1]. Any compression or resistance localized between the bladder neck and the urethral meatus negatively affects the bladder emptying, the sexual activity and thus the quality of life [2]. The most common cause of bladder outlet obstruction in men is benign prostatic hyperplasia (BPH), leading eventually to lower urinary tract symptoms [3]. These functional symptoms are consecutive to bladder structural changes, from *detrusor* hypertrophy to fibrosis, described as bladder remodeling [4]. Hypoxia is one of the stress factors that arises in the remodeled *detrusor* muscle. Hypoxia induces compensatory mechanisms through pathways involving, for example, vascular endothelial growth factors (VEGF) for neoangiogenesis [4]. Moreover, urothelium overgrowth and smooth muscle hypertrophy might be driven by hypoxic *vegf* upregulation during bladder outlet obstruction [5]. Bladder remodeling also impairs mitochondrial function, as evidenced by a decrease in enzymes of the respiratory chain and an increase in anaerobic metabolism, together with the generation of reactive oxygen species (ROS). In turn, if ROS production exceeds antioxidant systems, oxidative stress results in lipid peroxidation of (among others) mitochondria. If sustained, persistent depressed energy production contributes to impaired *detrusor* contractility [6]. Bladder remodeling also involves a significant decrease in *detrusor* innervation, directly associated with outflow tract obstruction and reversible after obstruction relief [7]. It has been suggested that after a damage, nerve regeneration might be delayed by the dysfunction of glial acidic fibrillary protein (GFAP) [8], which is a cytoskeletal protein present in Schwann cells and upregulated after axonal injury [9].

In men with prostate disorders leading to urethral sub-obstruction, medical therapies aim to inhibit prostate growth (5α-reductase inhibitors) and prostate smooth muscle contraction (α1-adrenergic receptor blockers, phosphodiesterase inhibitors) or to alleviate bladder contractions (anti-muscarinic drugs) while prostate surgery is elected if severity or progression of the troubles are no longer tolerable [3,10,11]. The goal of current treatments is to alleviate lower urinary symptoms, but no therapy exists to restore the bladder *detrusor* muscle once irreversible morphological changes have occurred. In such cases, regenerative therapy based on stem cells might represent an attractive option to repair the fibrotic dysfunctional bladder. In men, clinical studies performed on patients with partial bladder outlet obstruction have obtained encouraging results with autologous stem cells injected into the bladder wall under cystoscopic guidance [12,13,14].

Animal models are required to study the role of stem cells in bladder outlet obstruction, but current models suffer from limitations. Indeed, most models are short-term lethal models based on rodents and lack intermediary histological observations [15]. Moreover, to study the potential therapeutic value of stem cells, research protocols should emphasize on the study of the pathophysiology of the disease and the mode of action of stem cells, on determining an optimal source of stem cells, as well as a safe and efficient stem cells quantity to administer [16]. In this context, the chemokine C-C motif ligand 2 (CCL2) has been identified as overexpressed by human urinary bladder smooth muscle cells when exposed to elevated hydrostatic pressures [17]. Moreover, in mice, it has been demonstrated that CCL2 expression was increased in obstructed bladders compared to control group, and it has been suggested that this overexpression may be related to mesenchymal stem cells recruitment when administered as a treatment of bladder dysfunction consecutive to outlet obstruction [18].

Contrary to rodents, and because of similarities between men and dogs regarding lower urinary tract anatomy, the canine species could represent a valuable animal model to study the effects of bladder outlet obstruction on bladder *detrusor*. Indeed, bladder outlet sub-obstruction is also encountered in male dogs but most commonly related to urolithiasis or urinary tract neoplasia rather than BPH, which also occurs in aging dogs. Urethral sub-obstruction in dogs corresponds to the reduction of urinary flow during urination attempt, leading to dysuria, stranguria, pollakiuria and even hematuria [19]. If left untreated, consequences of bladder outlet obstruction in dogs can lead to urinary incontinence, retention, bladder atony, severe urinary tract infections or even to euthanasia.

The main goals of this pilot study were to evaluate the feasibility of an acquired model of urethral sub-obstruction in male dogs, as well as the feasibility of the use of autologous mesenchymal stem cells in this acquired model. Therefore, the objectives were twofold:

1. To induce a progressive urethral sub-obstruction in two dogs and to describe the functional and structural modifications of the urinary bladder over time: at different time scales, we aimed to describe bladder functional changes through urodynamic and clinical assessments, as well as morphological changes through macroscopic (ultrasonography), microscopic (histology, transmission electronic microscopy (TEM), immunohistochemistry) and molecular (RT-qPCR, bulk RNA sequencing, oxidative stress markers) assessments.
2. To describe, in one of those two dogs, the functional and structural changes observed following the administration of autologous mesenchymal stem cells.

We hypothesized that (1) functional and structural modifications of the *detrusor* muscle would be observed in both dogs and would be comparable to the changes described in men with urethral sub-obstruction in order to validate this acquired canine model and that (2) the reversibility of those changes after release of the sub-obstruction would be more noticeable after administration of autologous mesenchymal stem cells.

## 2. Materials and Methods

### 2.1. Dogs

Two sexually intact male Beagle littermates were included. They were born and housed at the local animal facilities. Animal housing and care as well as experimental procedures were approved by the Ethical committee of Animal Use of the University of Liège (reference number 1813). Outside experimental sessions, they were housed with conspecifics, maintained under standard kennel conditions, and benefited from regular human interaction and play periods with other dogs. Due the frequency of experimental procedures, the two dogs were monitored at least three times per week throughout the study. Prior to each experiment, physical examination of each dog was performed. Urine sample was obtained weekly via free catch collection for urinalysis including a dipstick test (Urispec Plus VET 10 Plus urinalysis strips, Henry Schein Inc, Melville, NY, USA), specific gravity measurement with a manual refractometer (Euromex microscopen, Arnhem, the Netherlands) and Diff-Quik-stained cytologic examination. Urine sample was submitted to a commercial laboratory (Synlab Laboratoire Collard, Liège, Belgium) for bacterial culture if infection was suspected on the basis of bacteriuria with evidence of phagocytosis by polymorphonuclear neutrophils on cytologic evaluation. If the bacterial culture was positive, the dog was temporarily excluded from the protocol and treated according to the antibiogram until a negative culture was obtained. Jugular venous blood samples were collected for each dog every other second week for creatinine and blood urea nitrogen analysis Catalyst Dx, Idexx Laboratories Inc, Westbrook, ME, USA).

For each surgical procedure (subcutaneous tissue harvesting, bladder biopsies, telemetric device implantation, artificial urethral sphincter (AUS) placement and retrieval), each dog was placed under general anesthesia and received midazolam (Midazolam, Mylan Institutional Inc., Canonsburg, PA, USA, 0.2 mg/kg), methadone (Comfortan, Dechra Veterinary Products Ltd., Shrewsbury, UK, 0.2 mg/kg, IV), propofol (Diprivan, Astra Zeneca, Cambridge, UK, 2 to 4 mg/kg, IV) and cefazolin (Cefazolin, Sandoz Inc., Princeton, NJ, USA, 20 mg/kg, IV). A cuffed endotracheal tube was placed, and anesthesia was maintained with isoflurane in oxygen. Postoperatively, each dog received cefazolin (20 mg/kg, IV, every 8 h) and methadone (0.2 mg/kg, IV, every 4 h) for 24 h as well as carprofen (Rimadyl, Zoetis Inc., Kalamazoo, MI, USA, 2 mg/kg, IV the first day then orally during 4 days, twice a day). Carprofen was not administered concomitantly to stem cells administration.

### 2.2. Study Design

In both dogs, the urethral sub-obstruction was progressively induced by gradual repletion of an AUS. The AUS of 14 mm cuff width and 10 mm lumen diameter was placed around the urethra, caudally to the prostate, during a caudal laparotomy approach. The size of the sphincter was selected according to manufacturer recommendations (Norfolk Vet Products, Skokie, IL, USA). The maximal AUS capacity (AUS repletion to obtain a full spreading of the AUS cuff) was assessed during the surgery, and the device was left totally empty at the end of the procedure to allow a progressive and gradual AUS repletion throughout the study phases.

**Figure 1:**
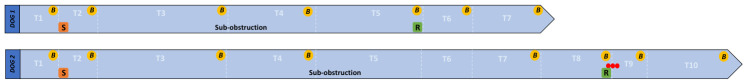
Study timeline of both dogs. S: AUS placement; R: AUS release; B: bladder biopsy; red dot: ADMSC administration. In both dogs, T1 corresponds to the phase before sub-obstruction. The progressive sub-obstruction period (progressive AUS insufflation) corresponds to phases T2 to T5 in dog 1 and to phases T2 to T8 in dog 2. The process of sub-obstruction was stopped by AUS removal, before the beginning of T6 in dog 1 and before the beginning of T9 in dog 2. The study proceeded after AUS removal until T7 in dog 1 and T10 in dog 2.

Data were recorded before and during the establishment of the sub-obstruction as well as after the sub-obstruction release. The study was divided into successive phases (Table 1): T1 to T7 for dog 1 and T1 to T10 for dog 2. In both dogs, T1 corresponded to the phase before sub-obstruction. The progressive sub-obstruction was performed by incremental repletion of the AUS cuff. Successive increases of 10% of the maximal AUS capacity were performed over months. The progressive sub-obstruction period corresponded to phases T2 to T5 in dog 1 and to phases T2 to T8 in dog 2. The process of sub-obstruction was stopped by AUS removal once dysuria was noticed (trouble to start the micturition necessitating several attempts before starting, slow or irregular urine flow, dribbling at the end of micturition). The AUS was removed before the beginning of T6 in dog 1 and before the beginning of T9 in dog 2; consequently the phases of maximal sub-obstruction were T5 in dog 1 and T8 in dog 2. In dog 2, the time of AUS removal was then considered as day 1 for autologous PKH26-adipose derived mesenchymal stem cells (ADMSCs) administration, and a bolus of 20 × 10^6^ ADMSCs was injected over 5 min in its cephalic vein. The same bolus was repeated on days 4 and 8 after AUS removal. Dog 1 did not receive ADMSCs. The study proceeded after AUS removal until T7 in dog 1 and T10 in dog 2. A simplified timeline of the study is presented in Figure 1.

At each phase of the study, bladder biopsies, urodynamic telemetric recordings, urethral profilometry, ultrasonography of bladder and kidneys, measurements of post-voiding residual urine volume, and blood samplings were scheduled in each dog. Table 1 summarizes the details of data collection across the various phases of the study for both dogs.

### 2.3. Autologous Adipose-Derived Mesenchymal Stem Cells

A strip of 5 × 1 × 1 cm subcutaneous adipose tissue of dog 2 was obtained under general anesthesia, during the same procedure as telemetric device implantation, and was placed in phosphate-buffered saline (PBS). Adherent and fibroblast-like cells were obtained from subcutaneous adipose tissue explants as previously described [20]. These cells were characterized in order to satisfy the minimal criteria of the International Society for Cell Therapy [21,22], which have already been described in dogs [23,24]. Briefly, cells at their third passage were incubated with antibodies anti-CD29 (phycoerythrin conjugated mouse monoclonal IgG1, BioLegend, San Diego, CA, USA), anti-CD44 (phycoerythrin conjugated mouse monoclonal IgG1, BioLegend, San Diego, CA, USA), anti-CD90 (phycoerythrin conjugated rat monoclonal IgG2b kappa, eBioscience, Thermofischer Scientific, Waltham, MA, USA) and anti-CD45 (fluorescein conjugated rat monoclonal IgG2b kappa, eBioscience, Thermofischer Scientific, Waltham, MA, USA). After one hour, cells were rinsed before analysis by flow cytometry. In addition, the cell multipotency was assessed by their ability to differentiate into adipogenic, chondrogenic, and osteogenic cells: briefly, differentiation was induced according to manufacturer’s guidelines (Stempro differentiation kits, Gibco, ThermoFischer Scientific, Waltham, MA, USA) and following specific periods of culture (7, 14 and 21 days, respectively) in appropriate differentiation media. Ultimately, adipogenic differentiation was checked by Oil Red O staining (Sigma-Aldrich, Saint Louis, MO, USA) for the presence of lipid droplets in the cytoplasm. The osteogenic differentiation was assessed by Alizarin Red S staining for calcium deposits in extracellular matrix (Sigma-Aldrich, Saint Louis, MO, USA). For chondrogenic differentiation, the proteoglycan-rich extracellular matrix was detected by Alcian Blue staining.

After isolation and characterization and a fourth passage, aliquots of ADMSCs were prepared and stored in liquid nitrogen. For subsequent use, ADMSCs were labelled with a red fluorescent membrane dye, PKH26, at low passage (less than 6) according to manufacturer recommendations (PKH26 Red Fluorescent cell Linker Kit, obtained from Sigma Aldrich Saint Louis, MO, USA). Briefly, frozen cells were thawed, cultured until 90% confluence in 2 flasks T175, and then trypsinized. The detached ADMSCs were at passage 5 and washed by a serum-free medium and resuspended in 1 mL of dilution buffer from the manufacturer’s labelling kit. The cell suspension was mixed with an equal volume of the labelling solution containing 4 × 10^−6^ M of PKH26 in the dilution buffer and incubated for 5 min at room temperature. After the termination of the reaction by adding 2 mL fetal bovine serum (Gibco, ThermoFischer Scientific, Waltham, MA, USA), cells were washed 3 times with the Dulbecco’s modified Eagle’s medium (Gibco, ThermoFischer Scientific, Waltham, MA, USA) and observed by fluorescent microscopy. A fraction of PKH26 labelled cells was maintained in culture during 10 days to monitor their long-term stability and viability.

Living cells were counted after a Trypan blue staining (Invitrogen, ThermoFischer Scientific, Waltham, MA, USA) to ensure a viability rate superior to 90% before injection.

### 2.4. Urodynamic Telemetric Study

**Figure 2:**
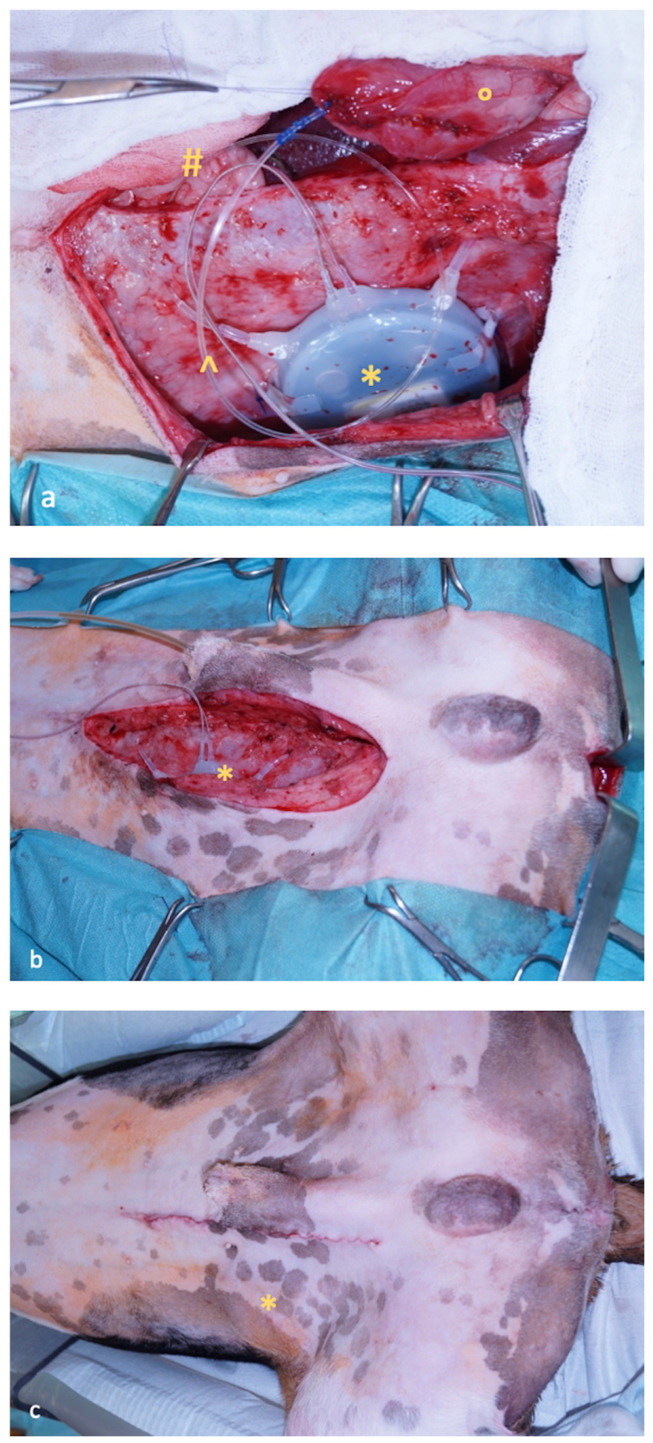
Surgical implantation of the telemetric device. Dog’s head is to the left, dog’s tail is to the right, and dog is in dorsal recumbency. (**a**): A caudal laparotomy has been performed to access the bladder (°) for placement of the first fluid-filled catheter (^) as well as the abdominal cavity for the placement of the second fluid-filled catheter (#). The transmitter body (*) has been inserted into a sub-cutaneous pocket created on the right flank of the dog. (**b**): Abdominal wall has been sutured, and the transmitter body (*) was secured to it before closure of the sub-cutaneous pocket. (**c**): The sub-cutaneous pocket has been closed, and the transmitter body (*) can be visualized under the skin.

The telemetric system is composed of a transmitter, a receiver, a data acquisition system and uroflowmetry system. The transmitter (Physio Tel Multiplus TL11M3-D70-PCTP, DSI) is composed of two biopotential leads (not used in this study) and two fluid-filled catheters with a thin-walled tip detecting sensitive pressure changes which are transferred to the pressure sensor located in the transmitter body. The transmitter body was surgically placed into the subcutaneous space in the left flank. One catheter was placed through the apical bladder wall with the extremity inside the bladder cavity to measure the bladder pressure, while the second catheter was left free inside the abdominal cavity to measure the abdominal pressure (Figure 2).

The data were digitized in the electronic module and sent via radio frequency waves to a remote receiver (RMC-1 Physio Tel, DSI) placed on the lateral wall of a metabolic cage. The receiver interfaced with the data exchange matrix (Dataquest ART Data Exchange Matrix, DSI) connected to an ambient pressure recorder and to the Dataquest Acquisition and Analysis System ART (DQ ART 3.1 Gold CM, DSI). The telemetric study protocol was divided into successive phases according to the progressive increase of the urethral sub-obstruction, starting before the AUS placement and finishing after the AUS retrieval. A washout period of 2 weeks was observed when a bladder biopsy was performed, between two phases of sub-obstruction.

Telemetric tests were repeated every week until the end of the study, except during the washout periods. Both dogs underwent telemetric urodynamic testing three times a week during a four-hour period. To force diuresis during this timeframe, 0.5 mg/kg of furosemide was injected intramuscularly at the beginning of each telemetric testing. During telemetric testing, each dog was provided with *ad libitum* food and water.

### 2.5. Urethral Pressure Profilometry

Urethral pressure profiles (UPP) were performed before harvesting the bladder biopsy. Anesthesia was induced with a bolus of propofol (6 mg/kg, IV) and maintained with a continuous rate IV infusion of propofol (maximum dose, 20 mg/kg/h). A light depth of anesthesia characterized by a central eye position and presence of a palpebral reflex and jaw tone without movement was maintained during testing. Three successive UPP measurements were performed as previously described [25,26], and the mean of the 3 measurements was reported for each urodynamic variable.

### 2.6. Ultrasonographic Study

**Figure 3:**
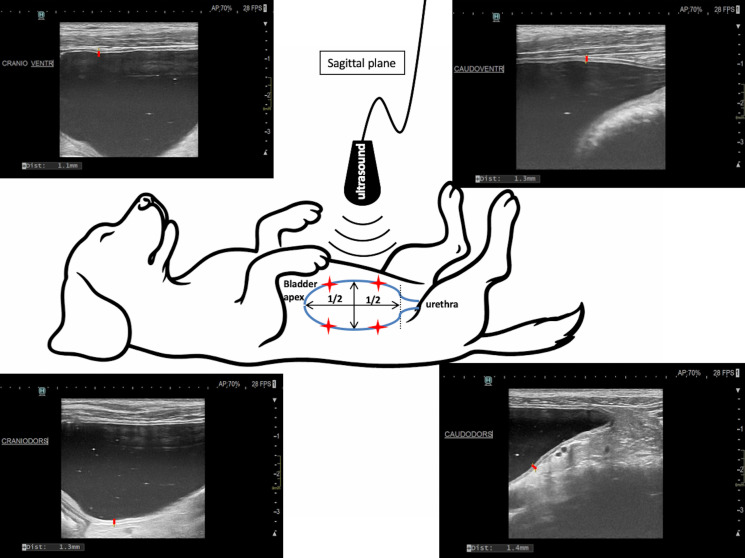
Ultrasonographic measurement of bladder wall thickness. Red stars on the drawing of the bladder correspond to the red dots on the ultrasound images and illustrate where the measurements were performed on the sagittal plane of the bladder. Top left: cranio-ventral location; top right: caudo-ventral location; bottom left: cranio-dorsal location; bottom right: caudo-dorsal location.

In both dogs, ultrasonography of the kidneys was performed every week to measure both renal pelvis and check for the absence of renal pelvis dilation, potentially indicating establishment of an acute urethral sub-obstruction rather than a progressive. Monthly, the bladder wall thickness was measured in both dogs, under standard bladder repletion: the bladder was emptied through urethral catheterization, and then 4 mL/kg of sterile NaCl was injected slowly. The thickness of the bladder wall was recorded at 4 points on a long-axis sagittal view of the bladder, 2 dorsally and 2 ventrally (Figure 3).

### 2.7. Post-Voiding Residual Urine

Monthly, post-voiding residual urine was collected in both dogs by aseptic urethral catheterization, and the volume was recorded. To facilitate and optimize spontaneous bladder emptying prior to residual urine collection, both dogs were walked on a leash for 10 to 15 min outdoors over various substrates (grass, asphalt, and woodland areas).

### 2.8. Samples

Bladder wall samples were obtained from both dogs at each phase of the study for histology, transmission electronic microscopy (TEM), immunohistochemistry, reverse transcriptase quantitative PCR (RT-qPCR), and RNA sequencing. Each bladder sample was performed through a full thickness bladder biopsy performed with a caudal laparotomy approach. The location of the biopsy site was recorded in the surgery report. The elected zone for the successive bladder biopsies was chosen away from the location of a previous biopsy (based on surgery report), as well as the presence of a scar and or epiploic adhesion.

Blood samples were obtained at each phase of the study for oxidative stress status assessment.

#### 2.8.1. Histological Assessment and Transmission Electronic Microscopy of Bladder Samples

Bladder tissue biopsies were fixed in 4% paraformaldehyde and paraffin-embedded and sectioned at 5 μm before analysis. The sections were stained with hematoxylin–eosin (HE) to observe the general morphology of the bladder, and Masson’s trichrome staining was performed to evaluate the extent of tissue fibrosis. The sections were evaluated in a non-blinded manner under a light microscope (Olympus BX51, Tokyo, Japan). On Masson’s trichrome-stained sections and using QuPath-0.5.1-x64 software [27], the total tissue area was manually delimited and measured using the polygon annotation tool, as well as the connective tissue areas located (i) between the urothelium and the innermost muscular layer and (ii) between the serosa and the outermost muscular layer. For each of these regions (sub-urothelial and sub-serosal), the connective tissue area was expressed as a percentage of the total tissue area.

Other bladder samples were immediately placed in 2.5% glutaraldehyde (glutaraldehyde 25% EM grade (Sigma Aldrich, Saint Louis, MI, USA) diluted in ultrapure water) in 0.1 M cacodylate buffer (Sigma Aldrich, Saint Louis, MI, USA), pH 7.2, at 4 °C and were cut into 1 mm^3^ pieces. They were then placed back in fresh fixation medium for at least 2 h at 4 °C. They were post-fixed with 1% osmium tetroxide (osmium tetroxyde 2% (Electron Microscopy Sciences, Morgantown, PA, USA) diluted in ultrapure water) in 0.1 M cacodylate buffer for 1 h, and then they were dehydrated in a series of rinses with increasing concentrations (30, 50, 70, 90, 100, 100, 100) of ethanol (ethanol EM grade, Sigma Aldrich, Saint Louis, MI, USA). An intermediate bath of epoxypropane (Electron Microscopy Sciences, Morgantown, PA, USA) was performed before the resin impregnation phase. The samples were then embedded in two parts of epoxypropane and one part of an Epon resin (Electron Microscopy Sciences, Morgantown, PA, USA) mixture, then 1:1 and 1:2 mixtures for 1 h each, and then overnight in pure Epon resin. Then, they were oriented in a mold with fresh Epon resin. Finally, they were incubated in a 60 °C oven for 48 h for resin polymerization. The samples were sectioned at 90 nm and stained with 0.5% uranyl acetate (Taab Laboratories Equipment Ltd., Aldermaston, UK) and lead citrate (VWR, Avantor, Radnor, PA, USA). Sections were observed on a Zeiss EM910 microscope (Zeiss, Oberkochen, Germany).

#### 2.8.2. RNA Extraction, Transcriptome and Gene Analysis

Bladder tissues from both dogs before AUS placement, at AUS removal and at the final biopsy were submitted to RNA sequencing. Bladder samples were placed into RNA later (Ambion, Thermo Fisher Scientific, Waltham, MA, USA USA). Samples were refrigerated at 4 °C for up to 24 h and then stored at −80 °C until further processing. Total RNA was extracted using a modified TRIzol protocol (Invitrogen, ThermoFischer Scientific, Waltham, MA, USA), in which, after the separation step, the aqueous phase containing RNA was transferred to NucleoSpin^®^ RNA kit extraction columns (Macherey-Nagel, Hamburg, Germany) and RNA extraction was continued, according to the manufacturer’s recommendations.

Total RNA quality control (QC) was performed using the 5200 Fragment Analyzer (Agilent Technologies, Santa Clara, CA, USA). Six samples (100 ng of total RNA, RQN score > 8) were processed using the Illumina Stranded Total RNA Prep, Ligation with Ribo-Zero Plus protocol (Illumina Ref kit 20040529). Library profiles were assessed on the QIAxcel Advanced system (QIAGEN) using a DNA screening cartridge. Final dual-indexed libraries were quantified by qPCR with the KAPA Library Quantification Kit including the Illumina standard (Roche reference 07960140001), then pooled equimolarly. Sequencing was performed on an Illumina NovaSeq 6000 sequencer on an S4 flow cell, generating paired-end 150-cycle reads at a sequencing depth of 50 million paired-end reads per sample. Raw sequencing data were demultiplexed using sample-specific indexes, filtered for quality, and converted into Fastq files for downstream analysis, using the bcl2fastq v2.20.0 software.

Sequencing data quality was assessed using the FastQC tool for individual reports per sample and with MultiQC to generate a report comprising all samples from a project.

Using the open bioinformatic tool RaNA-seq [28], samples were quantified with the Salmon algorithm [29] setting the following parameters: paired-end, default parameters. Due to the absence of biological replicates per time point, differential expression statistics were not performed. In each dog, log2 fold changes based on transcripts per million (TPM) normalized expression values were calculated for exploratory analysis between the initial time point and consecutive time points. A heat map was plotted to appreciate the pattern of regulation for selected genes (*ccl2*, *ccr2*, *vegf*, *gfap* and *hgf*).

#### 2.8.3. Assessment of CCL2, CCR2, GFAP, VEGF and HGF mRNA Expression in Bladder Samples by Reverse Transcriptase Quantitative Polymerase Chain Reaction

*β-actin* was selected as the reference housekeeping gene for normalization of target gene expression, and its primer sequences were described elsewhere [30,31]. The primer sequences for *ccl2* were described elsewhere [32], as well as the primers for *ccr2* [32], *gfap* [33], *vegf* [34] and *hgf* [35]. The primers sequences are reported in Table 2.

The PCR reactions were performed using a Luna Universal One-Step RT-qPCR Kit (Sigma Aldrich, Saint Louis, MI, USA) and contained 10 μL of Mix Luna, 5 μL of water UPure, 1 μL of enzyme from the kit, 1 μL of F primer and 1 μL of R primer. A total of 2 μL of RNA was added for a final volume of 20 μL. Samples were diluted at 10 ng/μL. The cycling conditions were as follows: 50 °C for 20 min and 95 °C for 5 min and then 45 cycles of 95 °C for 30 s and 60 °C for 30 s (for *β-actin*, *vegf-a*, *vegf-d* and *hgf*) or 64 °C for 30 s (for *vegf-b*, *vegf-c*, *ccl2*, *ccr2* and *gfap*) and a final extension at 72 °C for 30 s, using a QuantStudio™ 1 Real-Time PCR System (Thermofischer Scientific, Waltham, MA, USA). The specificity of the products obtained at the end of the PCR was confirmed by melting curve analysis. For each sample and time point, reactions were performed in duplicate and mean Ct values were calculated. The relative expression of each target gene was normalized to *β-actin* by calculating ΔCt values (Ct_target_ − Ct_β-actin_). Normalized expression levels were then expressed as 2^−ΔCt^, providing a log2-based measure of gene expression relative to the reference gene. In each dog, log2 fold changes were calculated between the initial time point and consecutive time points.

#### 2.8.4. CCL2, CCR2, GFAP and VEGF Immunohistochemistry on Bladder Samples

Fresh bladder tissue biopsies were rapidly frozen in OCT resin (Leica Biosystems, Deer Park, IL, USA) and stored at −80 °C. Cryosections of 10 μm were prepared using a Leica CM3050s cryostat. The sections were first fixed in acetone at 4 °C and then dried. After rehydration in PBS, the tissues were incubated with 0.03% hydrogen peroxide (peroxidase block, Agilent Technologies, Santa Clara, CA, USA) for 10 min to inhibit endogenous peroxidase activity and subsequently washed with PBS. To reduce non-specific staining, sections were blocked with 0.25% casein in PBS (Protein Block, Agilent Technologies, Santa Clara, CA, USA) for 10 min. The stainings (Table 3) were performed at room temperature in a wet chamber with 1 h incubation for the primary antibody, three 5 min rinses with PBS, and a 30 min incubation for the secondary antibody. At each staining round, negative controls were systematically performed by omitting the primary antibody for each secondary antibody used. After washing with PBS, immunoreactivity was revealed using 3-amino-9-ethylcarbazole (AEC) as chromogenic substrate (ImmPACT^®^ AEC Substrate Kit, Peroxidase, Vector Laboratories, Newark, CA, USA).

Sections were subsequently washed in distilled water and counterstained with Mayer’s hematoxylin for 2.5 min. Finally, the slides were washed with water and mounted with a commercial mounting medium (Glycergel mounting medium, Agilent Technologies, Santa Clara, CA, USA), scanned, and evaluated in a non-blinded manner using QuPath-0.5.1-x64 software [27] for positive cell counting. Briefly, on each slide, after color vector specification, the brush tool was used to manually define the muscular and urothelial areas. The remaining unclassified area was defined as “stroma”. Then, automatic cell detection tool was run using standard parameters, except for nucleus parameters sigma (1 μm), minimal area (15 μm^2^), and maximal area (200 μm^2^) and for threshold intensity (0.35). Finally, classification of the positive cells was made by creating a single measurement classifier with channel filter set on DAB, detection in the cell (DAB OD mean), positivity and negativity above and below threshold, respectively, and threshold being set between 0.15 and 0.25.

### 2.9. Oxidative Stress Status

Venous blood samples were collected from 8 a.m. to 9 a.m. in vacutainer tubes containing Na heparin or EDTA according to the tested parameter. Both dogs were fasted at least 12 h before blood sampling. External jugular venipuncture was performed with a 21-gauge needle, and blood samples were collected in the appropriate tubes. Part of whole blood was removed for reduced and oxidized glutathione and glutathione peroxidase determination. Then, both tubes were centrifuged at 1500× *g* during 10 min. A total of 500 μL of plasma EDTA were immediately added to 500 μL metaphosphoric acid (10%) for vitamin C stabilization and then frozen on dry ice. Another aliquot of 1 mL plasma EDTA was added to 5 μL butylhydroxytoluene at a concentration of 2 mg/mL in ethanol for the measurement of isoprostanes and then frozen on dry ice. The rest of EDTA plasma and serum were then discarded into separated tubes for analysis of vitamin E, myeloperoxidase, copper, zinc, and selenium and frozen on dry ice. All samples were then stored at −80 °C until analysis of oxidative stress biomarkers, performed according to protocols described elsewhere [36,37,38,39].

### 2.10. Data Interpretation

All results presented in this article are descriptive in nature; accordingly, they should be interpreted with due caution.

The following variables were measured or calculated from the 4 h telemetric continuous recordings: maximal and mean abdominal pressure (Pabdo_max_, Pabdo_mean_); threshold volume (fluid volume during micturition); and threshold *detrusor* pressure and mean and maximal *detrusor* pressure (Pdet_th_, Pdet_mean_, Pdet_max_, respectively, and calculated with the following formula: *detrusor* pressure = bladder pressure − abdominal pressure). Threshold parameters were calculated or measured during micturition. Compliance (C) was calculated with the following equation: C = (Vth − V_0_)/(Pblad_th_ − Pblad_0_) where Vth is threshold volume; V_0_ and Pblad_0_ are urinary bladder volume and pressure at the start of the recording, respectively; and Pblad_th_ is threshold pressure. Urinary flow (F) was calculated with the following formula: Flow = Vth/duration of micturition. Duration of micturition was measured in seconds. Urethral resistance was approximated with the following equation: Resistance = Pdet_mean_/F^2^, adapted from Buzelin and Glémin [40]. For each variable and for each study phase, a mean value was calculated from all the telemetric recordings of the corresponding phase.

The following variables were measured or calculated from the UPP measurements: maximum urethral pressure (MUP), maximum urethral closure pressure (MUCP, the difference between MUP and bladder pressure), integrated pressure (IP, area under the urethral functional profile) and length before maximum urethral pressure (LbMUP). Definitions were in accordance with those of the International Continence Society [41]. In the present study, 2 other variables were described to better assess the impact of the AUS on the initial part of the urethral pressure profile: LbMUP_AUS_ was defined as the profile length before the maximal urethral pressure induced by the presence of the AUS, and MUCP_AUS_ was defined as the MUCP at the profile length corresponding to the location of the AUS. These 2 variables were recorded before AUS placement, at AUS removal and at the final timepoint of the study.

The mean bladder wall thickness was calculated based on the measurements of the bladder wall at the 4 locations performed during each ultrasonographic assessment, for each study phase and in both dogs.

To obtain preliminary molecular insight into changes associated with bladder outlet obstruction and to explore the potential differences following ADMSC administration, we descriptively compared gene expression profiles of bladder tissues from both dogs before AUS placement, at AUS removal and at the final biopsy. We examined the expression level of several factors related to angiogenesis (*vegf*), neural filaments (*gfap*) or cellular chemotaxis (*ccl2*, *ccr2*) [42,43,44]. For these selected genes, we completed our observations with a RT-qPCR analysis and added *hgf* gene as an antifibrosis marker. Fold changes obtained by RT-qPCR and RNA sequencing were reported. Finally, we examined these data alongside protein expression data assessed by immunohistochemistry.

For the immunohistochemistry, in both dogs, QuPath-0.5.1 software [27] was used to assess the percentage of positive cells, for each marker (CCL2, CCR2, GFAP and VEGF), in 3 histological compartments (smooth muscle compartment, stroma and urothelium) at 3 study phases (before AUS placement, at AUS removal and at the final biopsy), based on the number of positive cells counted on 3 different slides.

## 3. Results

### 3.1. Dogs

The two dogs of this pilot study were 3 years old at the beginning of the study and 5 years old (dog 1) and 6 years old (dog 2), respectively, at the end of the study. Complete blood count, creatinine and blood urea nitrogen results were within the respective reference ranges for both dogs throughout the study. During the study, bacterial urine cultures revealed the presence of Pseudomonas aeruginosa twice in both dogs, during initial urethral sub-obstruction. Treatment was based on microbial susceptibility (marbofloxacin (Marbocyl, Vetoquinol, 4 mg/kg once a day), for 15 days and was associated to suspension of the experiment until urine culture turned negative.

In dog 1, dysuria was observed after 9 months of 75% AUS cuff repletion. In dog 2, dysuria was observed after 12 months of 115% AUS cuff repletion, obtained by exploiting the elasticity of the silicone beyond its nominal filling volume.

No clinical abnormalities or adverse events were observed, except for transient self-limiting hematuria following bladder biopsies (less than 1 day of duration), and no humane endpoints were reached. At study completion, both dogs returned to their normal kennel life without restriction or change in housing or activity.

### 3.2. Autologous Adipose-Derived Mesenchymal Stem Cells

**Figure 4:**
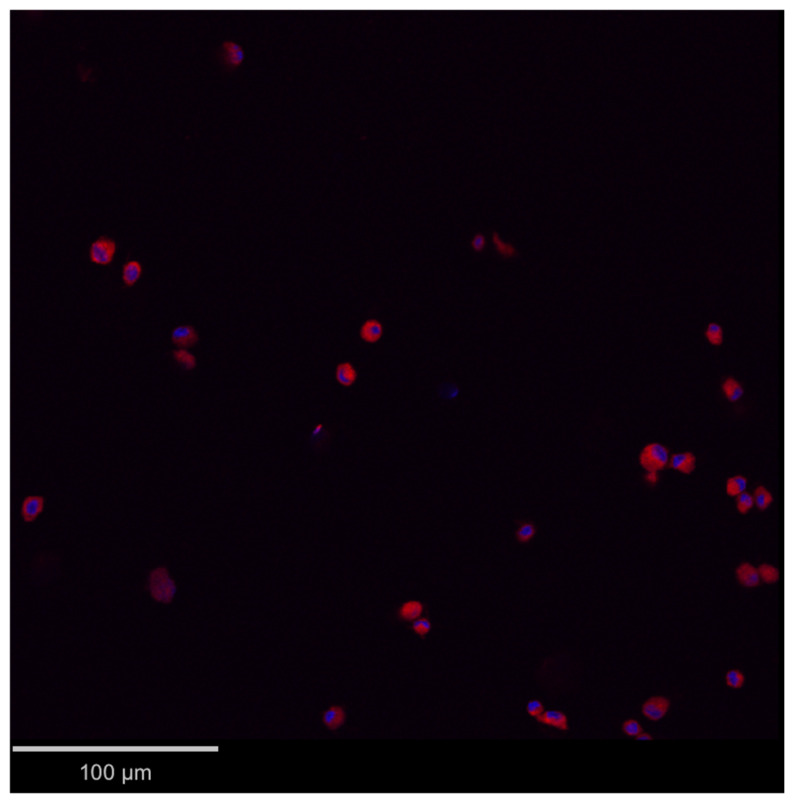
PKH26-labelled ADMSCs in a bladder wall full thickness biopsy of dog 2 at T9 (2 weeks after AUS removal and ADMSC injection). DAPI staining for nucleus localization (blue), overlaid with the same, red-fluorescent picture to identify PKH26-labelled ADMSCs.

Subcutaneous adipose tissue sampling was uneventful. Then, the isolation and culture methods allowed the harvesting, growing and long-term cryo-conservation of phenotypically characterized ADMSCs to enable 3 autologous administrations to dog 2, each containing 20 × 10^6^ viable cells. Mesenchymal stem cell positive markers were expressed (CD44, CD29 and CD90) while the negative marker was not present (CD45). The cell multipotency was demonstrated by their ability to differentiate into adipogenic, chondrogenic and osteogenic cells. PKH26-labelled ADMSCs were effectively observed in a bladder wall full thickness biopsy of dog 2 at T9 (Figure 4).

### 3.3. Urodynamic Telemetric Study

The surgical implantation of the telemetric devices was uneventful in both dogs. A self-limiting seroma was observed in both dogs postoperatively at the level of the subcutaneous pocket and resolved spontaneously within 10 days.

**Figure 5:**
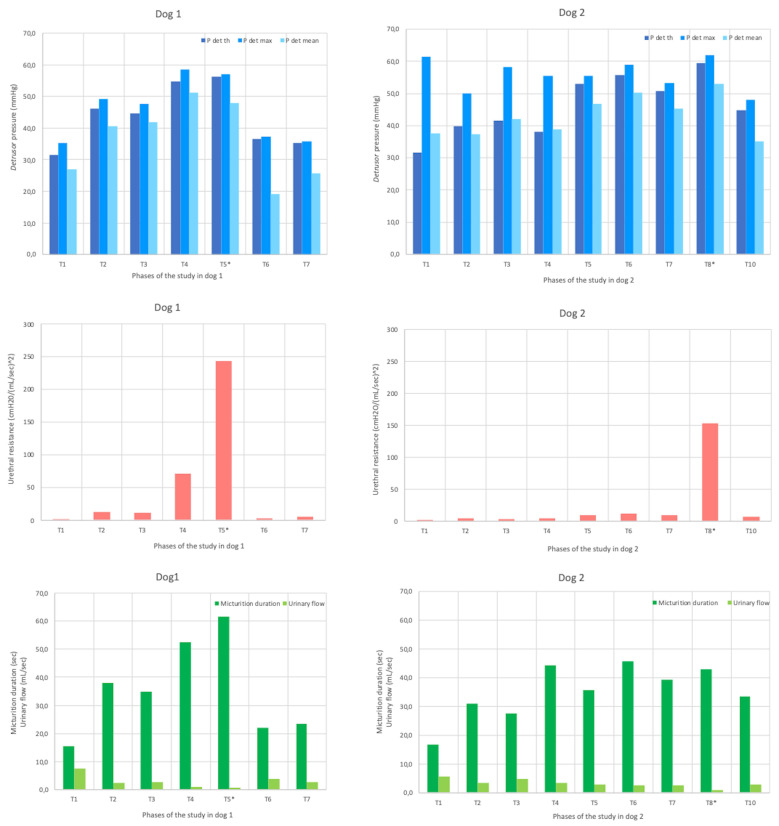
Evolution of detrusor pressures, urethral resistance, micturition duration and urinary flow in both dogs, according to the periods of the study. In each dog, the period of maximal sub-obstruction (T5 in dog 1 and T8 in dog 2) is highlighted by a star. P det th: threshold detrusor pressure; P det max: maximal detrusor pressure; P det mean: mean detrusor pressure.

The evolution of *detrusor* pressure, urethral resistance, urinary flow and micturition duration in both dogs are presented in Figure 5.

Values of abdominal pressures and bladder compliance are presented in Table 4 for dog 1 and in Table 5 for dog 2.

In both dogs, the following telemetric parameters evolved similarly:

- Threshold *detrusor* pressure was minimal at the start of the study and maximal at the time of maximal sub-obstruction and then decreased after AUS removal;
- Urinary flow was maximal at the start of the study, decreased to minimal value at the time of maximal sub-obstruction, and then increased after AUS removal;
- Urethral resistance showed an opposite trend to urinary flow: it was minimal at the start of the study, then increased to maximal value at the time of maximal sub-obstruction, and finally decreased after AUS removal;
- Bladder compliance decreased during the sub-obstruction period, from baseline value at the start of the study, reached minimal value at the time of maximal sub-obstruction, and then increased after AUS removal;
- Micturition duration increased during the sub-obstruction, starting from minimal value at the beginning of the study, to reach maximal value either at the time of maximal sub-obstruction in dog 1, or during the sub-obstruction period in dog 2. After AUS removal, micturition duration decreased in both dogs.

Other telemetric parameters evolved differently between the two dogs:

- Maximal and mean abdominal pressures were
  ○ maximal at the start of the study in dog 1, whereas they were maximal at the time of maximal urethral sub-obstruction in dog 2;
  ○ minimal at the end of the study in dog 1 whereas the minimum was reached during the sub-obstructive period in dog 2.
- Maximal and mean *detrusor* pressures were maximal during the sub-obstruction period in dog 1 and at the time of maximal sub-obstruction in dog 2. In dog 2, they were minimal at the end of the study. In dog 1, Pdet_max_ was minimal at the start of the study, while Pdet_mean_ was minimal after AUS removal.

### 3.4. Urethral Pressure Profilometry

In dog 1 (Table 6), IP was minimal at the start of the study (735 cm*cmH_2_O). Once the process of sub-obstruction started, IP increased and remained elevated until the end of the study (1282 cm*cmH_2_O). Maximum urethral pressure and MUCP were minimal during the period of sub-obstruction (166 mmHg and 152 mmHg, respectively) and maximal after AUS removal (339 mmHg and 336 mmHg, respectively).

In dog 2 (Table 7), IP was minimal during the sub-obstruction period (608 mmHg) and maximal at the time of maximal sub-obstruction (1267 mmHg). It decreased after AUS removal. Maximum urethral pressure and MUCP were minimal at the end of the study (64 mmHg and 62 mmHg, respectively) and maximal at the time of maximal sub-obstruction (158 mmHg and 154 mmHg, respectively).

LbMUP varied throughout the study phases in both dogs (Table 6 and Table 7).

**Figure 6:**
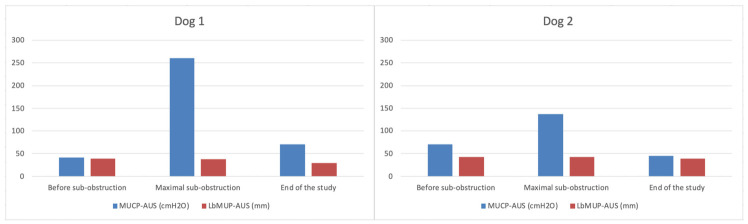
Values of LbMUP_AUS_ and MUCP_AUS_ in both dogs, demonstrating the effective urethral sub-obstruction at the AUS location (corresponding to stable LbMUP_AUS_), as illustrated by the increase in MUCP_AUS_ at the time of maximal sub-obstruction and decrease of MUCP_AUS_ at the same location before AUS placement and long-term after AUS removal.

MUCP_AUS_ and LbMUP_AUS_ values are shown in Figure 6 and demonstrate the effectiveness of the urethral sub-obstruction: in both dogs, values of MUCP_AUS_ increased between the start of the study (before AUS placement) and the time of maximal urethral sub-obstruction and then decreased at the end of the study (while AUS had been removed for several months). The steadiness of LbMUP_AUS_ throughout the study testifies that MUCP_AUS_ was measured at the same location along the urethra of each dog.

### 3.5. Ultrasonographic Study

In both dogs, measurements showed no acute increase of the right and left renal pelvis during the whole study. Mean bladder wall thickness varied from 1.3 mm before urethral sub-obstruction to 2.0 mm at maximal urethral sub-obstruction in dog 1. In dog 2, mean bladder wall thickness varied from 1.3 mm before urethral sub-obstruction to 1.8 mm at maximal urethral sub-obstruction (Table 8 and Table 9).

### 3.6. Post-Voiding Residual Urine Volume

In dog 1, maximal value of post-voiding residual volume was 4.1 mL/kg and was reached at maximal urethral sub-obstruction. The value before urethral sub-obstruction was 3.6 mL/kg, and it was 3 mL/kg at the end of the study. Minimal value during the process of urethral sub-obstruction was 1.7 mL/kg.

In dog 2, maximal value of post-voiding residual volume was 3.7 mL/kg and was reached at the phase of sub-obstruction which preceded the phase of AUS removal (corresponding to T7). The value before urethral sub-obstruction was 2.1 mL/kg, and it was 1.8 mL/kg at the end of the study. Minimal value during the process of urethral sub-obstruction was 1.2 mL/kg.

### 3.7. Histological and Transmission Electronic Microscopy Observations of Bladder Samples

**Figure 7:**
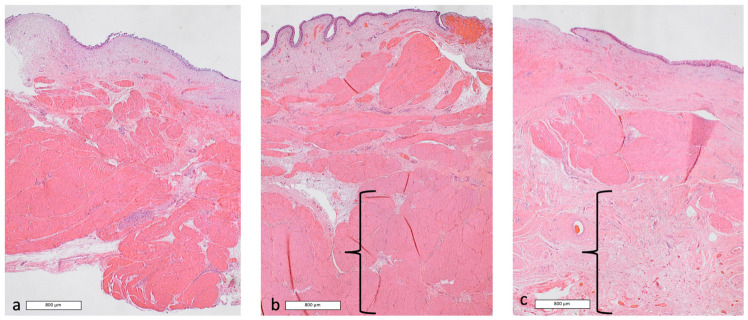
Histological assessment of bladder wall of Dog 1 (Hematoxylin eosin staining, magnification ×2). (**a**): before AUS placement; (**b**): at maximal sub-obstruction; (**c**): at final biopsy. Progressive loss of the plexiform organization of the muscular layer is highlighted by the brackets.

In dog 1, histological (HE) evaluation showed a normal bladder tissue structure except for the muscular layer, which lost its plexiform aspect and progressively appeared disorganized, from AUS removal (T6) until the end of the study (T7) (Figure 7).

**Figure 8:**
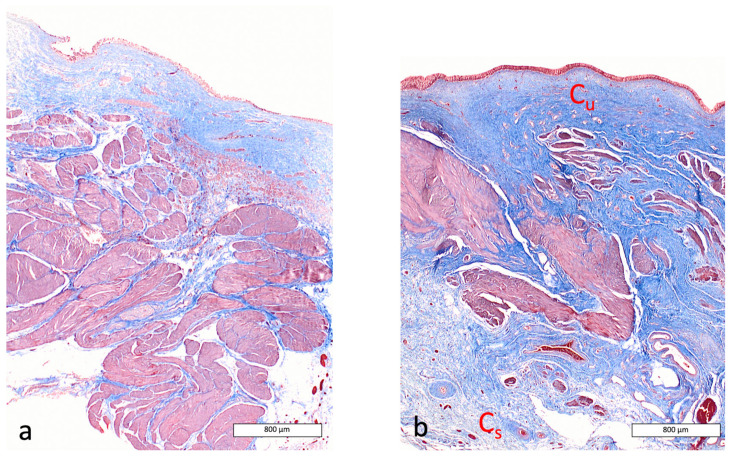
Comparison of the amount of connective tissue in the bladder wall of Dog 1 (Masson’s trichrome staining, magnification ×2). (**a**): before AUS placement; (**b**): at final biopsy. Cu and Cs highlight the stable and increased deposition of connective tissue, stable in the sub-urothelial layer and increased in the sub-serosal layer, respectively.

On Masson’s trichrome-stained sections, the sub-serosal layer exhibited a higher proportion of connective tissue in the final biopsy (T7, 7 months after AUS removal), accounting for 32% of the total tissue area versus 11.7% at T1. The proportion was more stable in the sub-urothelial layer (19% at T1 compared to 13.9% at T7) (Figure 8).

**Figure 9:**
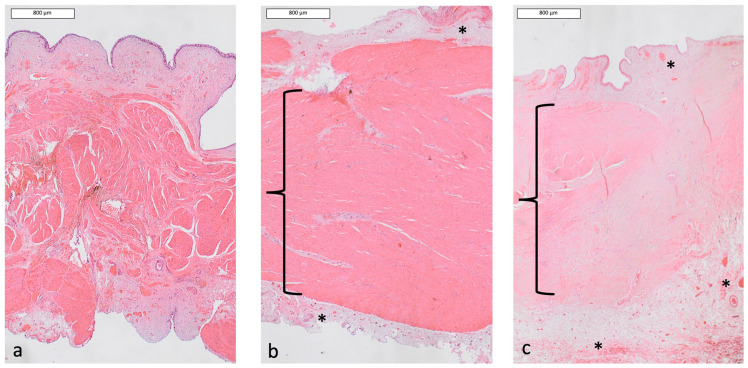
Histological assessment of bladder wall of Dog 2 (Hematoxylin eosin staining, magnification ×2). (**a**): before AUS placement; (**b**): at maximal sub-obstruction; (**c**): at final biopsy. Progressive loss of the plexiform organization of the muscular layer is highlighted by the brackets. Areas of hyperemia are highlighted by the stars.

In dog 2, in the urothelial layer, abundant presence of connective tissue in Masson’s trichrome staining and lymphoid infiltrate were observed in HE staining during the early period of sub-obstruction (second phase of sub-obstruction, corresponding to T3). At maximal urethral sub-obstruction, hyperemia was the main observation. In the final biopsy, this congestion was still noticed, as well as disorganization of the muscular layer (Figure 9).

**Figure 10:**
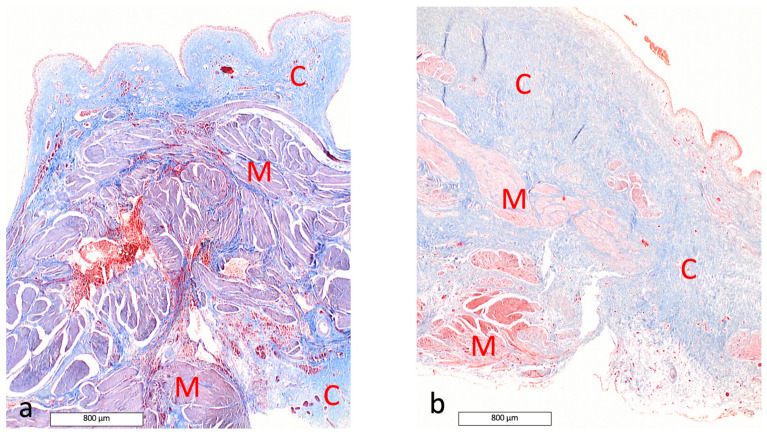
Comparison of the amount of connective tissue in the bladder wall of Dog 2 (Masson’s trichrome staining, magnification ×2). (**a**): before AUS placement; (**b**): at final biopsy. The evolution of the proportion of muscular tissue (M) versus connective tissue (C) is visible.

On Masson’s trichrome-stained sections, the sub-serosal layer exhibited a higher proportion of connective tissue in the final biopsy (T10, 5 months after AUS removal), accounting for 17.6% of the total tissue area versus 7.8% at T1. The proportion was also increased in the sub-urothelial layer (19.1% at T1 compared to 29.3% at T10) (Figure 10).

In both dogs, TEM observations of bladder samples revealed normal organelle morphology, especially normal mitochondrial morphology, throughout the study.

### 3.8. Reverse Transcriptase Quantitative Polymerase Chain Reaction Analysis and RNA Sequencing

**Figure 11:**
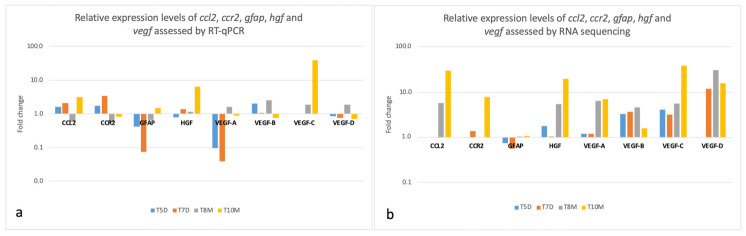
Relative expression levels of *ccl2*, *ccr2*, *gfap*, *vegf* and *hgf* assessed by RT-qPCR (**a**) and RNA sequencing (**b**) in both dogs. Expression data are presented as fold changes (log10 scale) calculated between the initial time point and consecutive time points. RT-qPCR data were normalized to β-actin, whereas RNA-sequencing data are expressed as transcripts per million (TPM). T5D: fold change during sub-obstruction in dog 1; T8M: fold change during sub-obstruction in dog 2; T7D: fold change after AUS removal in dog 1; T10M: fold change after AUS removal and ADMSC administration in dog 2.

For the selected genes, the evolution of the expression levels in both dogs is illustrated in Figure 11.

**Figure 12:**
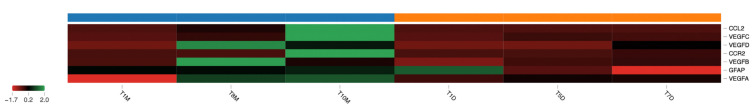
Heatmap of expression values (normalized as TPMs) of selected genes in each dog (Dog 1 in orange, Dog 2 in Blue) before AUS placement (T1M: Dog 2; T1D: Dog1), at maximal sub-obstruction (T8M: Dog 2; T5D: Dog 1) and at the final biopsy (T10M: Dog 2; T7D: Dog 1).

A heat map was generated to illustrate the pattern of expression obtained with RNA sequencing for *ccl2*, *ccr2*, *vegf*, *gfap* and *hgf* genes (Figure 12).

During the sub-obstructive period (from T1 to T5 in dog 1 and from T1 to T8 in dog 2), RT-qPCR analysis revealed consistent expression patterns in both dogs: *gfap* was downregulated (0.4 fold in dog 1 and 0.7 fold in dog 2) whereas *vegf-b* was upregulated (2.1 fold in dog 1 and 2.5 fold in dog 2). These patterns were also observed in RNA sequencing. During the same period, other marked gene expression changes were detected in at least one method included the following:

- in dog 1: upregulation of *ccl2* and *ccr2* (1.6 and 1.7 fold, respectively, in RT-qPCR),
- in dog 2: upregulation of *hgf*, *vegf-a*, *vegf-c* and *vegf-d* (5.4 fold in RNA sequencing for *hgf* and 1.6, 1.9 and 1.9 fold in RT-qPCR for *vegf-a*, *vegf-c* and *vegf-d*, respectively).

After AUS removal in both dogs and ADMSC administration in dog 2 (from T1 to T7 in dog 1 and from T1 to T10 in dog 2), RT-qPCR analysis revealed consistent expression patterns in both dogs: *ccl2* was upregulated (2.1 fold in dog 1 and 3.2 fold in dog 2), as well as *hgf* (1.4 fold in dog 1 and 6.5 fold in dog 2), whereas *vegf-d* was downregulated (0.8 fold in dog 1 and 0.7 fold in dog 2). These patterns were confirmed in RNA sequencing. Other marked gene expression changes detected in at least one method included the following:

- in dog 1: upregulation of *ccr2* and downregulation of *gfap* and *vegf-a* (3.4 fold, 0.1 fold and ~0 fold, respectively, in RT-qPCR),
- in dog 2: upregulation of *gfap* and *vegf-c* (1.5 fold and 38.8 fold, respectively, in RT-qPCR).

### 3.9. Immunohistochemistry

**Figure 13:**
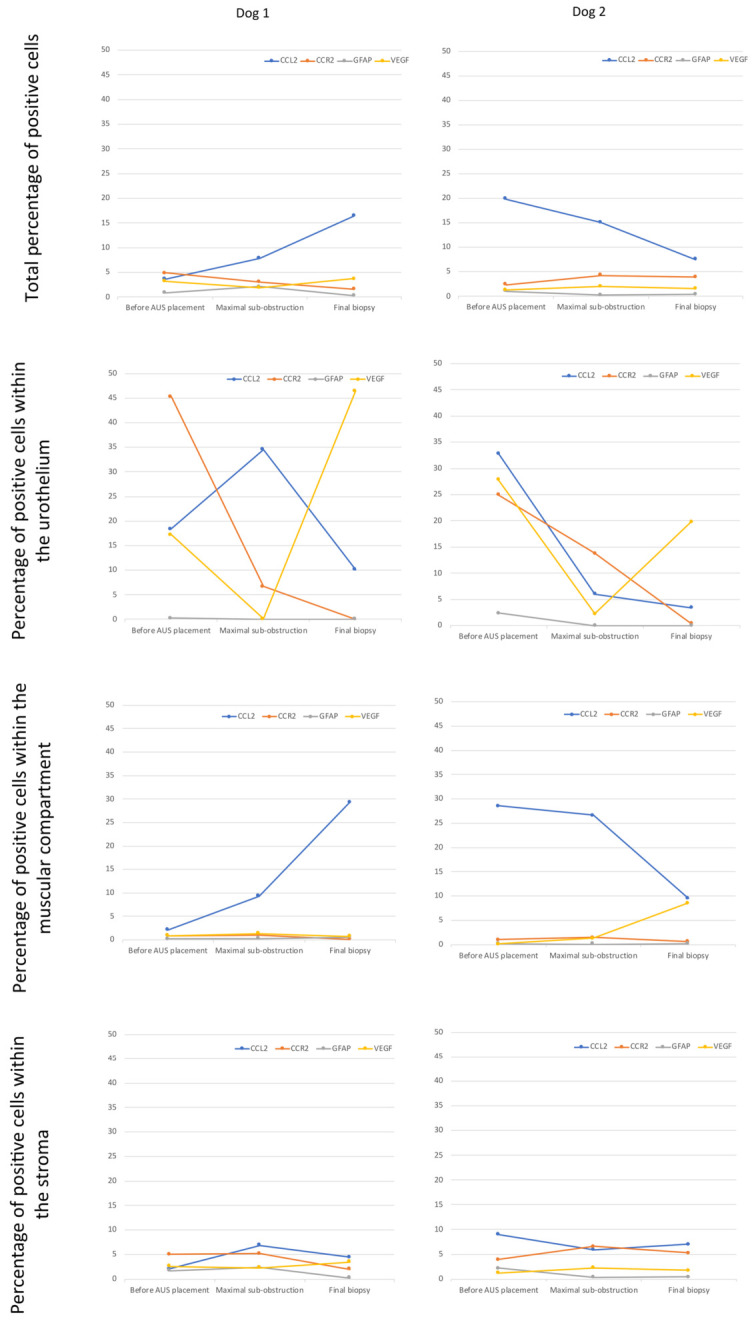
Percentage of positive cells for CCL2, CCR2, GFAP, and VEGF and their distribution within bladder urothelium, bladder muscular compartment and bladder stromal tissue over the study time. Left column: dog 1; right column: dog 2. Y-axis: percentage of positive cells; X-axis: study timepoints.

The percentage of cells positive for CCL2, CCR2, GFAP and VEGF, in both dogs, before AUS placement, at maximal urethral sub-obstruction and at final biopsy, is illustrated in Figure 13.

For CCL2, the total percentage of positive cells was noticeable throughout the study in both dogs. In the final biopsy, we observed a trend to increase of the percentage in dog 1 and to decrease in dog 2. Regarding the different compartments, positive cells were preferentially identified in the muscular layer and the urothelium in both dogs. In both dogs, the main change in the percentage of positive cells occurred at maximal sub-obstruction in the urothelial layer while it occurred at the final biopsy in the muscular compartment: increase in dog 1 and decrease in dog 2.

For CCR2, the total percentage of positive cells was moderate in both dogs throughout the study. In the urothelium, it was elevated before AUS placement, then decreased at the time of maximal sub-obstruction, and was null in the final biopsy. In both dogs, we observed almost no positive cells in the muscular compartment throughout the study and moderate but stable percentage of positive cells in the stromal compartment.

For GFAP, the total percentage of positive cells was low in both dogs before AUS placement and at maximal sub-obstruction in dog 1. It was null at the other times. Positive cells were only observed into the stromal compartment in dog 1 and in the urothelial and stromal compartments in dog 2.

For VEGF, the total percentage of positive cells was moderate in both dogs throughout the study. However, the percentage of positive cells in the urothelium was elevated in both dogs before AUS placement and in the final biopsy, and it was low at maximal sub-obstruction. In the muscular compartment, the percentage of positive cells was neglectable, except in the final biopsy of dog 2. In the stroma, the percentage of positive cells was low in both dogs throughout the study.

### 3.10. Oxidative Stress Status

**Figure 14:**
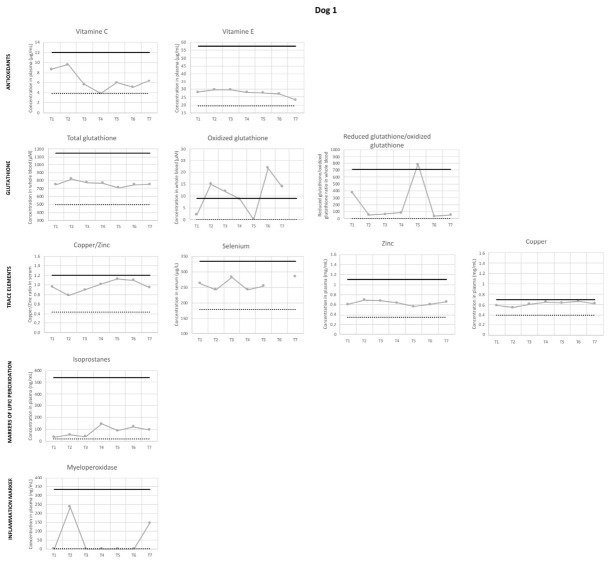

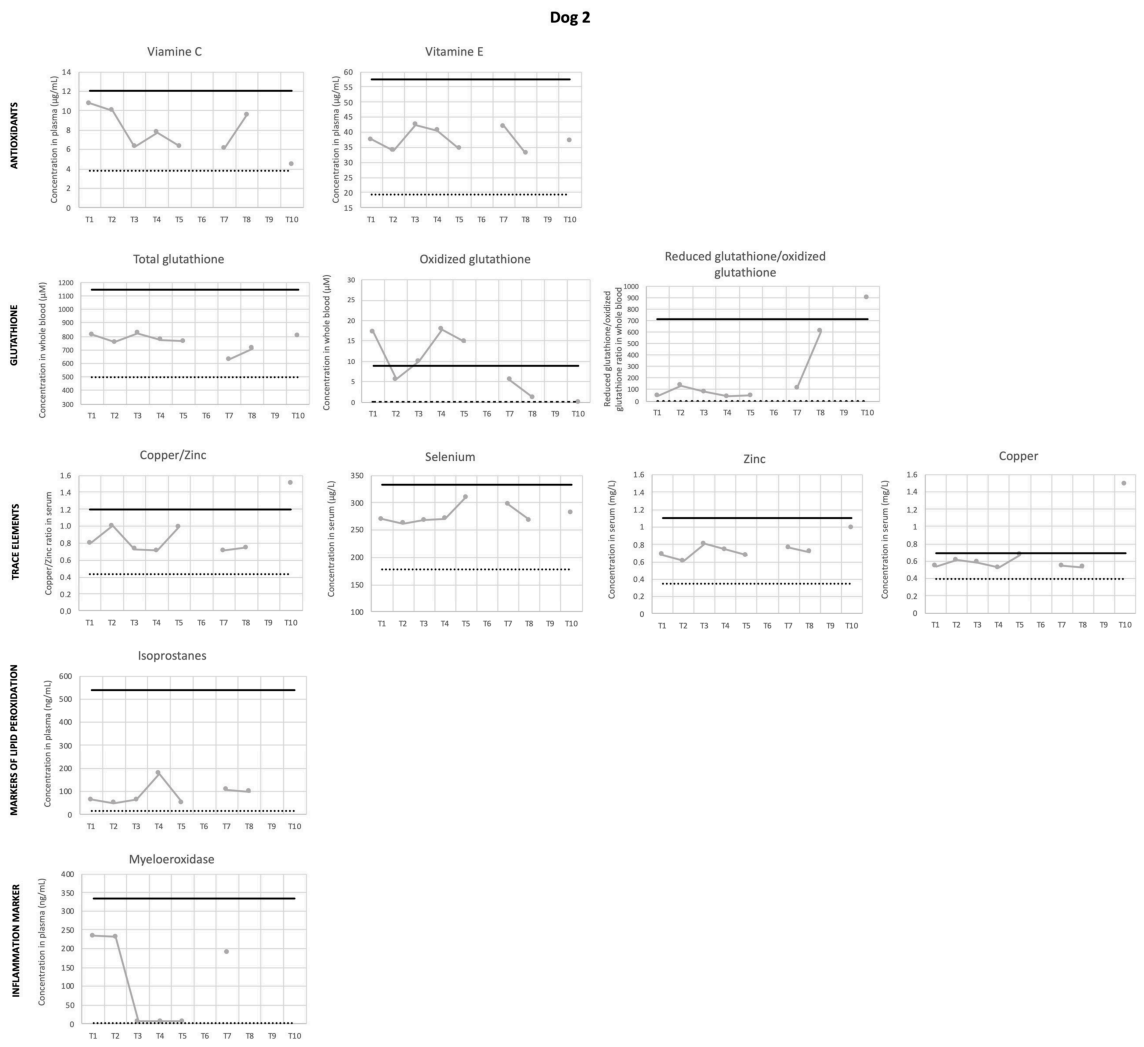
Oxidative stress parameters in both dogs according to the study phases. For each parameter, dark line and dotted line illustrate upper and lower physiological limits, respectively, described in healthy Beagle dogs [37].

Figure 14 shows the results of the measurements of the different oxidative stress parameters in both dogs. For each parameter, upper and lower limits are the 95% content physiological intervals previously reported in Beagle dogs [36].

In both dogs, all values of oxidative stress parameters were within normal limits throughout the study, except for oxidized glutathione, reduced glutathione/oxidized glutathione ratio and copper/zinc ratio, which showed values above the upper limit at specific timepoints (Figure 14).

In dog 1, a trend was observed throughout the study towards an increase in isoprostanes and copper/zinc ratio (driven by both a decrease in the antioxidant zinc and an increase in the prooxidant copper), along with a gradual decrease in vitamin C.

### 3.11. Summary of the Results

For both dogs, the main directional changes observed at the end of the sub-obstruction phase and at the end of the study across the four modalities (urodynamic, histological, molecular, and oxidative stress) are summarized in Table 10. The pre-obstructive period is not included, as it was considered the baseline condition.

## 4. Discussion

The objectives of this pilot study were to describe the functional and structural changes occurring in the urinary bladder secondary to a progressive sub-obstruction of the urethra induced by an AUS in two male dogs, as well as to describe those changes after release of the sub-obstruction and use of autologous ADMSCs in one of those dogs. The goal was to create changes comparable to those occurring in men with urethral sub-obstruction in order to potentially investigate the benefit of ADMSCs in restoration of bladder function.

Our results are descriptive in nature and should therefore be interpreted with caution. They indicate that the proposed canine model of acquired chronic and progressive urethral sub-obstruction, achieved using a suitably sized AUS, was appropriate to elicit clinical signs comparable to those reported in severely affected human patients. However, this occurred despite mild morphological alterations and only reversible urodynamic changes following sub-obstruction release. Notably, the development of severe dysuria and marked limitation of urinary flow precluded any further increase in the degree of urethral obstruction, as such progression would have led to an ethically unacceptable clinical situation for the two dogs. A decompensated state of the bladder was not achieved in this study, and the bladder of both dogs showed similar recovery following obstruction release. Therefore, this pilot descriptive study did not allow the identification of any potential effect of autologous stem cells administration on enhancing bladder recovery. However, in a pathological bladder setting, preparation and systemic administration of autologous stem cells appeared feasible and well tolerated in a single dog, in agreement with a previous report [45].

### 4.1. Functional Study

During the progression of the urethral sub-obstruction, both dogs showed a similar evolution of the functional urodynamic parameters, except for the abdominal pressure. At the peak of urethral resistance, abdominal pressure values barely decreased in dog 1 while they were elevated in dog 2, suggesting that both dogs behaved differently to oppose urethral impairment. In men with BPH, abdominal straining does not have a significant effect on urine flow rate [46]. We observed an increase in *detrusor* pressure during urethral sub-obstruction in both dogs, which is an indication of effective bladder outlet obstruction [4]. Regarding Pdet_th_, which is the pressure of the *detrusor* when micturition starts, it reached maximal values in both dogs when dysuria was noticed, at maximal degree of sub-obstruction. At that time, urethral resistance was more than 200 times higher than the baseline (pre-obstruction) value in dog 1 and almost 100 times higher than the baseline value in dog 2, and urinary flow reached its lowest level in both dogs. Since the removal of the AUS allowed a substantial decrease of the urethral resistance but did not allow restoration of urinary flow to values similar to the pre-obstructive period, even with stem cell-based adjunctive therapy, bladder contractility disorder was considered in both dogs. Indeed, in men with BPH, consideration of physical removal of bladder outlet obstruction through surgical treatment has to be balanced by the state of bladder contractility, since hypocontractility may lower clinical outcome postoperatively [47]. Decreased bladder contractility and consecutive increased post-voiding residual volumes are common observations in men with increased urethral resistance due to bladder outlet obstruction [48]. However, in the present study, post-voiding residual volume increased during urethral sub-obstruction but dropped below baseline values at the end of the study in both dogs. Therefore, an impairment of bladder contractility was excluded to explain the moderate urinary flow that was still observed in both dogs after AUS removal. Urethral fibrosis at the previous site of the AUS has also been suspected. However, since UPP did not show any persistence of elevated urethral pressures at the site of previous sub-obstruction after AUS removal, this hypothesis was also excluded.

At the time of maximal urethral sub-obstruction, bladder compliance was minimal in both dogs. Bladder compliance is a measurement of bladder distensibility and is reduced with bladder outlet obstruction [1], illustrating that a lower amount of urine is required into the bladder to increase the pressure by 1 cmH_2_O.

In the present study, functional impairment was assessed by the calculation of urethral resistance and the observation of micturition behavior of the dogs. In men, pressure-flow studies during micturition represent the gold standard method to confirm and quantify functional bladder outlet obstruction. Maximal urine flow is evaluated during micturition, and the corresponding *detrusor* pressure is measured. The relationship between pressure and urinary flow is then compared using standardized diagrams that determine the status of each patient [49,50]. Despite being precise and consistent, these invasive studies often cause discomfort to patients, so indirect methods are proposed to assess functional impairments. For example, in patients with lower urinary tract symptoms due to bladder outlet obstruction, predictive values of benign prostatic obstructive disorders can now be calculated using only non-invasive tests and help to guide therapies or further invasive diagnostic tests [51]. Imaging, such as MRI and ultrasonography, is of particular interest, due to the multiplicity of parameters that can be assessed to study bladder outlet obstruction, such as bladder wall and bladder position, urethra and peri-urethral structures such as prostate size and prostate relationship to the bladder, and others [52]. Ultrasonographic bladder wall thickness assessment is a way to measure non-invasively the hypertrophy of the *detrusor* secondary to outlet obstruction [53]. It has been shown that under specific measurement conditions, it is positively correlated with urodynamic parameters [54]. However, some limitations exist regarding the wide variability among studies, due to inconstant methodology (for example, bladder repletion, probe frequency, location of the measurements). Specifically, bladder wall thickness, *detrusor* wall thickness or ultrasound-estimated bladder weight are reported to assess bladder wall hypertrophy, but standardization between studies is still lacking [55]. In the present study, we used a standardized method of bladder repletion to perform reliable ultrasonographic assessments of the bladder wall thickness throughout the study. We observed a slight increase in bladder wall thickness at the end of the sub-obstructive period in both dogs, but decrease in bladder wall thickness was concomitant to sub-obstruction release only in dog 2. In both dogs, bladder wall thickness in the final biopsy was higher than initial value. However, due to the pilot nature of this study, we did not aim to perform statistical correlation with urodynamic parameters.

### 4.2. Structural Study

In the present study, structural changes supporting bladder wall morphological evolution during urethral sub-obstruction were barely observed. Given the large number of biopsies required by the experimental protocol for each bladder, and the numerous subsequent manipulations performed on each biopsy, particular care was taken in selecting the site of each biopsy, as described in the Materials and Methods, in order to obtain specimens of adequate size and free of scar tissue. However, considering the number of biopsies and the size of the canine bladder, the potential impact of repeated biopsies on both morphology and recovery should be acknowledged as a confounding factor. In dog 1, muscular bundle disorganization was observed from the end of the sub-obstruction period, as well as sub-serosal deposition of connective tissue after AUS removal. Whether these changes had a discreet role in the persisting modified functional values after AUS removal, compared to baseline values, is not established. In dog 2, structural changes were also subtle with temporary inflammatory reaction noticed during early urethral sub-obstruction (occurring 15 days prior to the first episode of bacterial isolation), hyperemia present at maximal urethral sub-obstruction and muscular bundle disorganization, as well as increased amount of sub-urothelial and sub-serosal connective tissue at the end of the study.

These differences between the two dogs might be related to the different expression of molecular signals during the study. Indeed, a differential pattern of global gene expression between the two dogs was observed when comparing RNA sequencing data for the selected markers, as well as across the different levels of expression, from genes to proteins. However, these exploratory results are based on only two dogs and need further investigation in a larger cohort.

In dog 1, regarding mRNA quantification, *ccl2* gene expression increased after AUS removal. In our study, an increased expression of CCL2 was also observed at the protein level in the final biopsy. Histological analysis revealed connective tissue deposition, as demonstrated by Masson’s trichrome staining. The involvement of CCL2 and its receptor CCR2 in profibrotic signaling pathways, associated with M2 macrophage polarization, has previously been reported [56], which may suggest a possible role for CCL2 in the structural changes observed in dog 1, although this remains speculative. Noteworthy, the increase in CCL2 immunostaining in dog 1 was mainly supported by the muscular compartment. Similar observation has been described in vitro, when muscular cells of the *detrusor* were submitted to oxidative stress induced by elevated hydrostatic pressure [17].

In dog 2, the levels of mRNAs for *ccl2* and *hgf* after AUS removal exhibited a similar pattern to that of dog 1, but with a greater amplitude, since the expression of these genes was markedly increased in the final biopsy, 3.2 and 6.5 fold changes in dog 2, respectively, compared to 2.1 and 1.4 fold changes in dog 1, respectively. However, in dog 2, the progression of mRNA expression of *ccl2* was opposed to the protein expression, the immunostaining of which decreased along the study. Indeed, in the final biopsy of dog 2, CCL2 detection with immunohistochemistry reached its lowest level, and the diminution was associated to a diminution of the expression of CCL2 in the muscular compartment. This pattern is opposite to what was observed in dog 1. Therefore, we can raise the hypothesis that this observation might be associated with post-transcriptional regulations secondary to ADMSC administration at the time of AUS removal in dog 2. Indeed, in rats with partial bladder outlet obstruction, it has been reported that stem cells may modify microRNAs involved in mRNA translation, thereby improving tissue repair in the stem-cell-treated group compared with untreated rats [57]. Under this hypothesis, a potential trend toward enhanced repair mechanisms could be further supported by the increase in mRNA levels of the *hgf* gene in the final biopsy of dog 2 (6.5 fold change) compared with dog 1 (1.4 fold change). However, only subtle morphological evidence of repair was perceived, with connective tissue deposition observed in the final biopsy of both dogs, involving two layers in dog 2 compared with only one layer in dog 1.

In the present study, a very low level of GFAP detection was observed in both dogs throughout the study. GFAP is a protein of the supporting cells of the nervous system. In the peripheral nervous system, it is expressed in Schwann cells after axonal damage [8,58]. Indeed, upregulation of Schwann cells’ repair-related phenotypic markers, such as GFAP, has been reported in a rat model of urethral sphincter denervation [59]. In the present study, the urethral sub-obstruction did not lead to bladder decompensation, since remodeling was limited. Thus, nerve damage did not occur, and the *gfap* gene was similarly and moderately downregulated during the sub-obstructive period in both dogs, and GFAP protein expression remained low as well. Absence of nerve damage may explain why consecutive *detrusor* underactivity and consistent fibrosis [60,61] were not observed.

Ultrastructure of the *detrusor* smooth muscle cells did not change throughout the study, in both dogs. More precisely, we did not observe alteration of the mitochondrial shape or density, which is observed and correlated with bladder outlet obstruction in men [62]. Indeed, it has been demonstrated that ROS increase after bladder outlet obstruction and cause mitochondrial lipid peroxidation, which in turn negatively affects energy production and *detrusor* contractility [6]. Since hypoxia and subsequent ischemia–reperfusion events are major triggers of oxidative stress and are typically associated with *vegf* upregulation, we also evaluated *vegf* expression. However, the lack of consistent regulation of *vegf* gene subtypes and the inconsistent VEGF protein expression do not support the presence of established hypoxia. Furthermore, in the present study, several observations suggest that oxidative stress status was balanced in both dogs, with absence of observed mitochondrial structural damage, absence of lipid peroxidation over the physiological upper limit and absence of impairment of *detrusor* contractility. However, during the initial phase of urethral sub-obstruction, oxidized glutathione, which is an endogenous peptide formed by the glutathione peroxidase enzyme and coupled with organic peroxides reduction, increased over upper physiological limit and decreased at the end of the sub-obstructive period to reach physiological interval. After AUS removal, oxidized glutathione increased again over upper physiological in dog 1, whereas it remained within physiological range in dog 2. Whether this observation can be related to ADMSC administration in dog 2 cannot be inferred by our results. However, a paracrine action of stem cells to balance oxidative stress status can be hypothesized, as proposed in a rodent model of bladder ischemia [63]. In our hypothesis, stem cells would contribute to the reduction of oxidative stress by enhancing glutathione redox mechanisms.

### 4.3. Limitations

The main limitation of our study is the use of only two dogs. We acknowledge that the absence of replication in the treatment condition prevents us from determining whether the observations arise from random variation or reflect a true treatment effect. Future work should incorporate appropriate replication to confirm the reliability of the observed trends. This study was meant to be a preliminary study, before considering a potential designed controlled study on more individuals. Indeed, because of the scarcity of comparable models in dogs, and even though BPH can naturally occur in this species unlike in rodents, validation of this proposed canine model was critical before any further extensive experiment. So, this study was designed as a longitudinal study of each dog. Furthermore, we also wanted to test the safety and feasibility of the regenerative treatment before potentially inducing severe bladder lesions in several dogs, to ensure that an experiment which would otherwise be unjustifiable, could be rendered ethically acceptable. Moreover, the use of littermates was intended to minimize inter-individual variability by taking advantage of their similar genetic background.

A second limitation is the repeated collection of bladder biopsies, which may constitute a confounding factor. Although this issue was anticipated during protocol development and particular care was taken at each sampling time point, it may nonetheless have introduced some bias into the results.

At this stage, the model cannot be considered directly translatable to humans and should be further refined to better reproduce human pathophysiology. Despite a long-lasting progressive urethral sub-obstructive period (1.5 year in dog 1 and 2.5 years in dog 2), significant functional and pathological structural alterations were not observed in our study, contrary to what is reported naturally in men [64] and experimentally in rodents [65]. Furthermore, certain data that might have helped to more accurately characterize the underlying molecular mechanisms were not collected, such as HGF immunohistochemical analyses as well as the precise identification of the cellular populations exhibiting immunohistochemical positivity, or the measurement of whole blood glutathione peroxidase activity. In men, bladder tissue remodeling, a consequence of chronic bladder outlet obstruction, leads to serious functional disorders that worsen initial obstructive symptoms. In the decompensated stage, voiding dysfunction leads to a further decline in quality of life [4]. It should be acknowledged that animal models have inherent limitations in explaining the complex relationship between disease, symptoms, and dysfunction [40], and several hypotheses may explain why our canine model was limited to reversible changes. Indeed, in men, the duration of the compensated phase depends on factors such as the patient’s age at the onset of lower urinary tract symptoms, the severity and nature of the obstructive disease, and the presence of aggravating conditions, such as vascular or metabolic disorders [66]. In the present study, the dogs were young and healthy at the onset of urethral sub-obstruction, which may offer opportunities for further refinement of the model.

Another limitation is the variability in the number of “effective” micturitions recorded across telemetric sessions and study phases, which limits accurate comparisons between them. Following AUS removal, both dogs exhibited a period lasting several weeks, during which normal micturitions alternated with varying degrees of urinary incontinence. This included involuntary urine leakage immediately before and after micturition, when the dog was not yet or no longer in position, as well as splitting of the urinary stream with intermittent pauses, rather than a single, continuous and straight stream. During these periods, only normal micturitions were included in the analysis, resulting in a reduced amount of telemetric data.

Telemetric urodynamics may be considered as a limitation, due to the potential loss of accuracy of the device along time. Indeed, at the time of telemetric device retrieval, vesical line incrustation was observed in the dogs of the present study. This observation has already been reported and does not necessarily compromise data accuracy [67]. Moreover, telemetry is considered as a valuable tool in urologic research, with a good repeatability in dogs, especially for the measurements obtained during the night [68], which was the case in our study since most of the recordings were performed in the evening. When associated to furosemide induced diuresis, it allows reproducible voiding patterns with no effect on urodynamic parameters. Indeed, at a dose four times higher than that used in our study, furosemide has been shown to significantly increase voiding frequency and volume per void, without affecting micturition pressure [69]. However, the potential impact of long-term administration of furosemide on our results cannot be ruled out.

Regarding mRNA expression, although absolute values differed between the two methods we used (RT-qPCR and RNA sequencing), the direction of regulation was consistent for *ccl2*, *ccr2*, *gfap*, and *vegf-b* genes in dog 1 and for *ccl2*, *hgf*, *gfap*, and *vegf-c* in dog 2. The discrepancies observed between the methods are attributed to the different normalization approaches applied in RT-qPCR and RNA sequencing. Using an additional housekeeping gene, such as *glyceraldehyde-3-phosphate dehydrogenase*, could have helped strengthen our analysis.

## 5. Conclusion

In this pilot study, our first hypothesis was rejected since we did not observe bladder decompensation secondary to diffuse fibrosis of the bladder *detrusor*, comparable to what is described in men with chronic bladder outlet obstruction. As such, the current proposed model cannot be validated as a canine model for urethral sub-obstruction. Consequently, our second hypothesis could not be validated either, as the reversibility of the functional and structural changes was mainly attributed to the sub-obstruction release (AUS removal) in both dogs.

Autologous ADMSCs constitute ethically acceptable regenerative therapies in both human and veterinary medicine. Furthermore, functional follow-up in awake subjects using urodynamic telemetry should be preferred whenever possible, as it allows studies to be conducted under conditions that more closely resemble the pathophysiological state.

Finally, we showed that the systemic administration of autologous ADMSCs was safely feasible in one dog. Refinement of the model in a control study may help to better assess the observations made in the treated dog of this study after sub-obstruction release. Indeed, whether stem cell administration helped to balance oxidative stress status by enhancing the glutathione redox buffer system and interfered with CCL2 signaling by reducing CCL2 protein synthesis remains to be confirmed.

Further investigation is required to refine this canine model of bladder outlet obstruction and to identify clear and durable changes within the bladder wall, prior to the objective validation of non-invasive regenerative medicine approaches for human bladder restoration.

## Figures and Tables

**Table 1:** Time schedules of the study for each dog.

DOG 1													
Procedure/Study Phase		T1		T2	T3		T4		T5		T6	T7	
		01/2020–02/2020		02/2020–04/2020	05/2020–11/2020		01/2021–03/2021		05/2021–09/2021		10/2021–01/2022	01/2022–04/2022	
**Urodynamic telemetric recording**		every week (days 1, 3, 5)		every week (days 1, 3, 5)	every week (days 1, 3, 5)		every week (days 1, 3, 5)		every week (days 1, 3, 5)		every week (days 1, 3, 5)	every week (days 1, 3, 5)	
**Percentage of AUS cuff repletion**		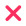 (no AUS)		0–60%	60–70%		75%		75%		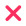 (no AUS)	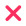 (no AUS)	
**Urethral sub-obstruction**		before obstruction		chronic progressive	chronic progressive		chronic progressive		maximal		0–3 months after AUS removal	3–7 months after AUS removal	
**Urethral pressure** **profilometry**		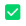 at the end of the period		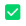 at the end of the period	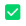 at the end of the period		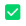 at the end of the period		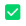 at the end of the period		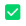 at the end of the period	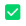 at the end of the period	
**Bladder ultrasonography**		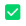 at the end of the period		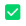 at the end of the period	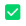 at the end of the period		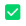 at the end of the period		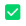 at the end of the period		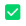 at the end of the period	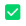 at the end of the period	
**Urine residual volume collection**		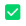 at the end of the period		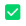 at the end of the period	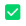 at the end of the period		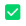 at the end of the period		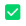 at the end of the period		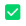 at the end of the period	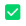 at the end of the period	
**Bladder wall full** **thickness biopsy**		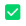 at the end of the period		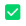 at the end of the period	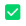 at the end of the period		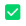 at the end of the period		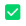 at the end of the period		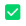 at the end of the period	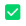 at the end of the period	
**Oxidative stress status (blood)**		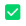 at the end of the period		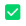 at the end of the period	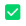 at the end of the period		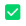 at the end of the period		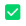 at the end of the period		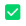 at the end of the period	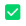 at the end of the period	
**Urinalysis**		weekly		weekly	weekly		weekly		weekly		weekly	weekly	
**Creatinine and blood urea nitrogen analysis**		every 2 other week		every 2 other week	every 2 other week		every 2 other week		every 2 other week		every 2 other week	every 2 other week	
**Kidney ultrasonography**		weekly		weekly	weekly		weekly		weekly		weekly	weekly	
**DOG 2**													
**Procedure/Study Phase**	**T1**		**T2**	**T3**	**T4**	**T5**		**T6**		**T7**	**T8**	**T9**	**T10**
	**01/2020–02/2020**		**02/2020–04/2020**	**05/2020–11/2020**	**01/2021–03/2021**	**05/2021–09/2021**		**10/2021–01/2022**		**01/2022–04/2022**	**05/2022–09/2022**	**09/2022–10/2022**	**10/2022–03/2023**
**Urodynamic** **telemetric** **recording**	every week (days 1, 3, 5)		every week (days 1, 3, 5)	every week (days 1, 3, 5)	every week (days 1, 3, 5)	every week (days 1, 3, 5)		every week (days 1, 3, 5)		every week (days 1, 3, 5)	every week (days 1, 3, 5)	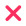	every week (days 1, 3, 5)
**Percentage of AUS cuff repletion**	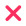 (no AUS)		0–60%	60–70%	75%	85–115%		115%		115%	115%	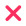 (no AUS)	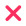 (no AUS)
**Urethral** **sub-obstruction**	before obstruction		chronic progressive	chronic progressive	chronic progressive	chronic progressive		chronic progressive		chronic progressive	maximal	2 weeks after AUS removal/ADMSCs injection	0.5–5 months after AUS removal
**Urethral pressure profilometry**	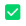 at the end of the period		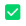 at the end of the period	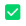 at the end of the period	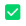 at the end of the period	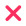		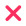		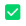 at the end of the period	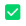 at the end of the period	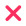	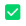 at the end of the period
**Bladder** **ultrasonography**	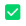 at the end of the period		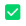 at the end of the period	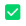 at the end of the period	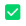 at the end of the period	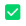 at the end of the period		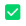 at the end of the period		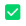 at the end of the period	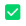 at the end of the period	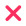	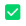 at the end of the period
**Urine residual** **volume collection**	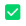 at the end of the period		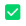 at the end of the period	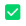 at the end of the period	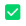 at the end of the period	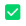 at the end of the period		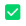 at the end of the period		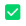 at the end of the period	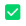 at the end of the period	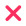	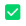 at the end of the period
**Bladder wall full thickness biopsy**	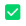 at the end of the period		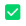 at the end of the period	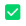 at the end of the period	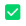 at the end of the period	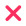		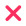		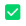 at the end of the period	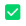 at the end of the period	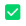 at the end of the period	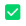 at the end of the period
**Oxidative stress status (blood)**	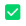 at the end of the period		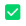 at the end of the period	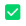 at the end of the period	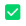 at the end of the period	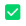 at the end of the period		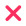		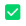 at the end of the period	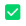 at the end of the period	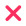	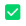 at the end of the period
**Urinalysis**	weekly		weekly	weekly	weekly	weekly		weekly		weekly	weekly	weekly	weekly
**Creatinine and blood urea** **nitrogen analysis**	every 2 other week		every 2 other week	every 2 other week	every 2 other week	every 2 other week		every 2 other week		every 2 other week	every 2 other week	every 2 other week	every 2 other week
**Kidney** **ultrasonography**	weekly		weekly	weekly	weekly	weekly		weekly		weekly	weekly	weekly	weekly

**Table 2:** Canine-specific primer sequences used for RT-qPCR evaluation of bladder samples from dogs.

Gene	Primers		Reference
*gfap*	F	AGATCCACGATGAGGAGGTG	Lim et al., 2017 [33]
	R	TCTTAGGGCTGCTGTGAGGT	
*hgf*	F	AAAGGAGATGAGAAACGCAAACAG	Spee et al., 2005 [35]
	R	GGCCTAGCAAGCTTCAGTAATACC	
*ccl2*	F	AAAGAGTCACCAGCAGCAAG	Riddell et al., 2022 [32]
	R	ATGGCTTTGCAGTTTGGGTT	
*ccr2*	F	TGTAAGTCATTCACGGGGCT	Riddell et al., 2022 [32]
	R	CTGAGGACTGCAGGAGGAAA	
*vegf-a*	F	GCTGCTGTAATGATGAGGGC	Lee et al., 2024 [34]
	R	CCCTTCCCCTTTCCTCGAAT	
*vegf-b*	F	TCTGACTGTGGAGCTCATGG	Lee et al., 2024 [34]
	R	TTCTTCCAGGGACATCTCGC	
*vegf-c*	F	GCCCAACATCAGTGCAAGAA	Lee et al., 2024 [34]
	R	TTGTTGCTGCTCCAAACTCC	
*vegf-d*	F	GAACAGCAGATTAGGGCAGC	Lee et al., 2024 [34]
	R	TGCCACTCCTCGTCTATGAC	
*β-actin*	F	GAGACCTGACCGACTACCT	Qiu et al., 2008 [30,31]
	R	GCTGCCTCCAGACAACAC	

**Table 3:** Antibodies used for immunostaining.

Protein	Primary Antibody	Secondary Antibody
CCR2	polyclonal rabbit anti CCR2 1/100 (Santa Cruz *)	HRP-Goat anti Rabbit IgG impress Vector ready to use
CCL2	polyclonal Mouse anti canine CCL2/JE/MCP 1 1/100 (R&D **)	HRP-Goat anti mouse IgG impress Vector ready to use
VEGF	monoclonal Mouse anti VEGF 1/50 (Santa Cruz)	HRP-Goat anti mouse IgG impress Vector ready to use
GFAP	polyclonal Rabbit anti GFAP 1/500 (Dako)	HRP-Goat anti Rabbit IgG impress Vector ready to use

* Santa Cruz Biotechnology (Dallas, TX, USA). ** R&D Systems (Minneapolis, MN, USA).

**Table 4:** Abdominal pressures and bladder compliance in dog 1 at each study phases. Pabdo_max_: maximal abdominal pressure; Pabdo_mean_: mean abdominal pressure. T1: phase before sub-obstruction. T2 to T5: progressive sub-obstruction period. T5: maximal sub-obstruction. T6 to T7: post-obstructive period.

DOG 1							
Variable/Study Phase	T1	T2	T3	T4	T5	T6	T7
	01/2020–02/2020	02/2020–04/2020	05/2020–11/2020	01/2021–03/2021	05/2021–09/2021	10/2021–01/2022	01/2022–04/2022
**Pabdo_max_ (mmHg)**	12.73	8.59	8.36	8.68	8.49	6.97	3.41
**Pabdo_mean_ (** ***mmHg*** **)**	8.26	6.30	6.11	5.69	5.12	4.56	−0.09
**Compliance (mL/cmH_2_O)**	3.10	1.80	1.90	0.90	0.60	6.70	2.20

**Table 5:** Abdominal pressures and bladder compliance in dog 2 at each study phases. P abdo max: maximal abdominal pressure; P abdo mean: mean abdominal pressure. T1: phase before sub-obstruction. T2 to T8: progressive sub-obstruction period. T8: maximal sub-obstruction. T9–T10: post-obstructive period. T9: no telemetric recording due to washout period.

DOG 2										
Variable/Study Phase	T1	T2	T3	T4	T5	T6	T7	T8	T9	T10
	01/2020–02/2020	02/2020–04/2020	05/2020–11/2020	01/2021–03/2021	05/2021–09/2021	10/2021–01/2022	01/2022–04/2022	05/2022–09/2022	09/2022–10/2022	10/2022–03/2023
**Pabdo_max_ (mmHg)**	1.76	0.41	−2.17	3.34	−10.13	−15.69	−15.74	5.74	/	−7.16
**Pabdo_mean_ (mmHg)**	−2.81	−3.77	−6.69	−5.08	−14.04	−19.3	−18.8	3.31	/	−12.79
**Compliance (mL/cmH_2_O)**	3.8	2.8	2.9	4.00	1.9	1.9	1.8	0.6	/	2.8

**Table 6:** Urodynamic variables in Dog 1 at each study phases. T1: phase before sub-obstruction. T2 to T5: progressive sub-obstruction period. T5: maximal sub-obstruction. T6 to T7: post-obstructive period.

DOG 1							
Variable/Study Phase	T1	T2	T3	T4	T5	T6	T7
	01/2020–02/2020	02/2020–04/2020	05/2020–11/2020	01/2021–03/2021	05/2021–09/2021	10/2021–01/2022	01/2022–04/2022
**MUP (cmH_2_O)**	207	226	166	181	313	339	206
**MUCP (cmH_2_O)**	207	217	152	172	299	336	205
IP (cm*cmH_2_O)	735	1276	1218	1026	1654	1399	1282
**LbMUP (mm)**	138	201	45	36	208	113	83

**Table 7:** Urodynamic variables in Dog 2 at each study phases. T1: phase before sub-obstruction. T2 to T8: progressive sub-obstruction period. T8: maximal sub-obstruction. T9–T10: post-obstructive period. T9: no telemetric recording due to washout period.

DOG 2										
Variable/Study Phase	T1	T2	T3	T4	T5	T6	T7	T8	T9	T10
	01/2020–02/2020	02/2020–04/2020	05/2020–11/2020	01/2021–03/2021	05/2021–09/2021	10/2021–01/2022	01/2022–04/2022	05/2022–09/2022	09/2022–10/2022	10/2022–03/2023
**MUP (cmH_2_O)**	107	130	120	137	/	/	79	158	/	64
**MUCP (cmH_2_O)**	99	125	106	129	/	/	74	144	/	62
IP (cm*cmH_2_O)	684	927	931	1094	/	/	608	1267	/	690
**LbMUP (mm)**	248	216	18,3	82	/	/	89	67	/	192

**Table 8:** Ultrasonographic study in dog 1. T1: phase before sub-obstruction. T2 to T5: progressive sub-obstruction period. T5: maximal sub-obstruction. T6 to T7: post-obstructive period.

DOG 1							
Variable/Study Phase	T1	T2	T3	T4	T5	T6	T7
	01/2020–02/2020	02/2020–04/2020	05/2020–11/2020	01/2021–03/2021	05/2021–09/2021	10/2021–01/2022	01/2022–04/2022
Mean renal pelvis measure (*Right: Left*, mm)	1.0:0.8	1.1:1.0	1.1:1.0	1.2:1.0	1.1:1.0	1.1:1.3	1.3:1.4
Mean bladder wall thickness (mm)	1.3	1.3	1.5	1.5	1.7	2.0	1.5

**Table 9:** Ultrasonographic study in dog 2. T1: phase before sub-obstruction. T2 to T8: progressive sub-obstruction period. T8: maximal sub-obstruction. T9–T10: post-obstructive period. T9: no telemetric recording due to washout period.

DOG 2										
Variable/Study Phase	T1	T2	T3	T4	T5	T6	T7	T8	T9	T10
	01/2020–02/2020	02/2020–04/2020	05/2020–11/2020	01/2021–03/2021	05/2021–09/2021	10/2021–01/2022	01/2022–04/2022	05/2022–09/2022	09/2022–10/2022	10/2022–03/2023
Mean renal pelvis measure (*Right: Left*, mm)	0.9:0.9	1.1:0.9	1.1:0.9	1.2:1.0	1.1:0.9	1.0:0.9	1.0:0.8	1.0:0.8	0.9:1.0	1.0:0.8
Mean bladder wall thickness (mm)	1.3	1.3	1.3	1.5	1.5	1.2	1.2	1.8	/	1.6

**Table 10:** Summary table. Pdet_th_: threshold *detrusor* pressure. IP: integrated pressure. Increase (**↑**) or decrease (↓) are relative to the maximal sub-obstruction period (urodynamics) or to the period before sub-obstruction (molecular study). “Elevated” refers to the upper limit of the 95% content physiological interval [36].

Study Period	Urodynamics		Histology		Molecular Study		Oxidative Stress	
	Dog 1	Dog 2	Dog 1	Dog 2	Dog 1	Dog 2	Dog 1	Dog 2
**Maximal sub-** **obstruction**	Maximal Pdet_th_ Maximal urethral resistance Minimum urinary flow Maximal IP Minimal bladder compliance		Disorganization of muscular layer		↓ *gfap* expression **↑** *vegf-b* expression		Elevated reduced glutathione/oxidized glutathione ratio	/
				Hyperemia	**↑** *ccl2* and *ccr2* expression **↑** CCL2 protein expression (urothelium + *detrusor*)	**↑** *hgf*, *vegf-a*, *vegf-c* and *vegf-d* expression ↓ CCL2 protein expression (urothelium + *detrusor*)		
**End of the study**	↓ urethral resistance **↑** urinary flow (not to baseline values) **↑** bladder compliance (not to baseline values)		**↑** connective tissue in sub-serosal layer	**↑** connective tissue in sub-serosal layer and in sub-urothelial layer	**↑** *hgf* expression **↑** *ccl2* expression ↓ *vegf-d* expression		Elevated oxidized glutathione	Elevated reduced glutathione/oxidized glutathione ratio
	Pdet_th_ back to baseline IP still elevated	Pdet_th_ not back to baseline IP back to baseline			**↑** *ccr2* expression ↓ *gfap* and *vegf-a* expression **↑** CCL2 protein expression (*detrusor*)	**↑** *gfap* expression ↓ *vegf-c* expression ↓ CCL2 protein expression (urothelium + *detrusor*)		

## Data Availability

The raw data supporting the conclusions of this article will be made available by the authors on request.

## Abbreviations

The following abbreviations are used in this manuscript:

BOO	Bladder outlet obstruction
AUS	Artificial urethral sphincter
RNA	Ribonucleic acid
mRNA	Messenger ribonucleic acid
ADMSCs	Adipose-derived mesenchymal stem cells
BPH	Benign prostate hyperplasia
VEGF	Vascular endothelial growth factor
ROS	Reactive oxygen species
GFAP	Glial acidic fibrillary protein
CCL2	Chemokine C-C motif ligand 2
TEM	Transmission electronic microscopy
RT-qPCR	Reverse transcriptase quantitative polymerase chain reaction
HE	Hematoxylin-eosin
CCR2	C-C chemokine receptor type 2
HGF	Hepatocyte growth factor
AEC	3-amino-9-ethylcarbazole
UPP	Urethral pressure profilometry
MUP	Maximal urethral pressure
MUCP	Maximum urethral closure pressure
IP	Integrated pressure
LbMUP	Length before maximal urethral pressure
Pdet	*Detrusor* pressure
Pabdo	Abdominal pressure
Pblad	Bladder pressure
V	Bladder volume
C	Bladder compliance
